# Multi-Strategy Improved Dung Beetle Optimization Algorithm and Its Applications

**DOI:** 10.3390/biomimetics9050291

**Published:** 2024-05-13

**Authors:** Mingjun Ye, Heng Zhou, Haoyu Yang, Bin Hu, Xiong Wang

**Affiliations:** 1 School of Information Science and Technology, Yunnan Normal University, Kunming 650500, China; 2 Department of Internet of Things and Artificial Intelligence, Wuxi Vocational College of Science and Technology, Wuxi 214028, China; 3 College of Engineering, Informatics, and Applied Sciences, Flagstaff, AZ 86011, USA; 4 Department of Computer Science and Technology, Kean University, Union, NJ 07083, USA; 5 School of Information Science and Engineering, Yunnan University, Kunming 650500, China

**Keywords:** dung beetle optimization algorithm, Latin hypercube sampling, mean differential variation, dimension-by-dimension optimization

## Abstract

The dung beetle optimization (DBO) algorithm, a swarm intelligence-based metaheuristic, is renowned for its robust optimization capability and fast convergence speed. However, it also suffers from low population diversity, susceptibility to local optima solutions, and unsatisfactory convergence speed when facing complex optimization problems. In response, this paper proposes the multi-strategy improved dung beetle optimization algorithm (MDBO). The core improvements include using Latin hypercube sampling for better population initialization and the introduction of a novel differential variation strategy, termed “Mean Differential Variation”, to enhance the algorithm’s ability to evade local optima. Moreover, a strategy combining lens imaging reverse learning and dimension-by-dimension optimization was proposed and applied to the current optimal solution. Through comprehensive performance testing on standard benchmark functions from CEC2017 and CEC2020, MDBO demonstrates superior performance in terms of optimization accuracy, stability, and convergence speed compared with other classical metaheuristic optimization algorithms. Additionally, the efficacy of MDBO in addressing complex real-world engineering problems is validated through three representative engineering application scenarios namely extension/compression spring design problems, reducer design problems, and welded beam design problems.

## 1. Introduction

Optimization is everywhere, be it engineering design, industrial design, business planning, holiday planning, etc. We use optimization techniques to solve problems intelligently by choosing the best from many available options [1]. At its core, it involves the quest for an optimal set of parameter values within specified constraints, aimed at either maximizing or minimizing system performance indicators [2]. Due to the involvement of many decision variables, complex nonlinear constraints, and objective functions, efficient methods are required for solving them. Traditional algorithms typically start from singularities and rely on gradient information [3]. However, many real-world optimization problems are often characterized as black-box problems, where specific expressions, gradient information, and derivatives are unknown [4]. Metaheuristic algorithms (MAs) are computational intelligence paradigms especially used for sophisticated solving optimization problems [5]. MAs present a promising avenue for tackling most real-world nonlinear and multimodal optimization challenges by offering acceptable solutions through iterative trial and error [6].

These algorithms are classified into evolutionary-based [7], physics-based [8], and swarm intelligence-based [9] categories. Evolutionary-based algorithms, rooted in natural selection and genetics, include genetic algorithm (GA)  [10] and differential evolution (DE)  [11]. GA evolves potential solutions by simulating natural selection and genetic mechanisms like replication, crossover, and mutation operations, gradually converging toward the optimal solution. DE mimics biological evolution to seek the optimal solution by leveraging differences among individuals in the population to guide the search direction and iteratively evolve towards the optimum. Physics-based algorithms allow each search agent to interact and move in the search space according to certain physical rules, with common algorithms, including simulated annealing (SA) [12], the gravitational search algorithm (GSA) [13], and the sine cosine algorithm (SCA) [14]. The SA algorithm simulates the physical annealing process, randomly exploring the solution space to find the global optimum solution, and utilizes a probability jump mechanism to avoid local optima, thus achieving global optimization. GSA is inspired by natural gravitational forces and object movements, aiming to find the global optimum by adjusting the positions of objects in the solution space for optimization search. Meanwhile, SCA utilizes the fluctuating properties of sine and cosine functions to generate random candidate solutions and, through an adaptive balance of exploration and exploitation stages, achieves global optimization search. Swarm intelligence (SI) algorithms are inspired by collective behaviors of social insects and animals [15]. Some classic swarm intelligence algorithms include particle swarm optimization (PSO) [16], ant colony optimization (ACO) [17], artificial bee colony (ABC) [18], grey wolf optimizer (GWO) [19], whale optimization algorithm (WOA) [20], Harris hawks optimization (HHO) [21], sparrow search algorithm (SSA) [22], and the slime mold algorithm (SMA) [23]. These algorithms exhibit characteristics of self-organization, adaptation, and self-learning and are widely applied across various domains [24].

The dung beetle optimization (DBO) algorithm [25] is a swarm intelligence algorithm, proposed in 2022, and has attracted considerable attention due to its well-optimized performance and unique design inspiration among a plethora of metaheuristic algorithms. DBO emulates various life behaviors of dung beetle populations, such as rolling balls, dancing, foraging, stealing, and reproduction, thereby constructing a novel optimization strategy. Experimental results demonstrate that DBO exhibits good performance in solving some classical optimization problems. Nevertheless, achieving desirable results when using the DBO algorithm to solve complex optimization problems remains a challenge. Specifically, the drawbacks of DBO are primarily evident in the following aspects: Firstly, during the initialization phase, the utilization of randomly generated populations may lead to an uneven distribution within the solution space, consequently restricting exploration and potentially trapping the algorithm in local optima. Secondly, the inclination toward greediness of the algorithm throughout the search process may precipitate premature convergence on local optima, disregarding the global optimum and resulting in suboptimal outcomes. Furthermore, akin to other swarm intelligence algorithms, when solving multi-dimensional objective functions, neglecting the evolution of specific dimensions due to inter-dimensional interference deteriorates convergence speed and compromises solution quality. As asserted by the “No Free Lunch” (NFL) theorem [26], every algorithm has its inherent limitations, and there is no one algorithm that can solve all optimization problems. Therefore, many scholars are dedicated to proposing new algorithms or improving existing ones to address various real-world optimization problems. This paper addresses the deficiencies and limitations of the original DBO algorithm by proposing a multi-strategy improved Dung Beetle Optimization algorithm (MDBO). The MDBO aims to enhance the global optimization capability of the original DBO by introducing multiple strategies, improving the convergence accuracy and speed of the algorithm. Then, the overall performance of the MDBO algorithm is validated through experiments across various aspects. Overall, the main contributions of this paper are as follows:

- The Latin hypercube sampling (LHS) initialization strategy replaces the original random initialization method of DBO to generate higher-quality initial candidate solutions.
- Introducing a mean difference mutation strategy enhances the capability of the algorithm to escape local optimal solutions by mutating the population.
- A strategy that combines lens imaging inverse learning with dimension-by-dimension optimization is proposed and applied to the current optimal solution to enhance its quality.
- The proposed MDBO algorithm is verified to outperform other classical metaheuristic algorithms in terms of performance by comparing the solution accuracy, convergence speed, and stability of the CEC2017 and CEC2020 functions, respectively.
- Further, MDBO was successfully applied to three real-world engineering optimization problems, validating its superior capability in solving complex engineering problems.

This paper is organized as follows. The basic dung beetle optimization algorithm is introduced in Section 2. The multi-strategy improved dung beetle optimization algorithm (MDBO) is proposed in Section 3 to address the shortcomings of the dung beetle optimization algorithm. In Section 4, the improved multi-strategy dung beetle optimization algorithm is experimentally compared with other algorithms in various aspects to verify the effectiveness of the improvement measures. Section 5 uses the improved algorithm in real-world engineering applications to further explore the practical applicability of the improved algorithm. Section 6 summarizes the full work.

## 2. The Basic Dung Beetle Optimization Algorithm (DBO)

The dung beetle optimization algorithm is inspired by the behaviors of dung beetles such as rolling, dancing, foraging, stealing, and reproduction. Four population renewal strategies are designed based on these behaviors.

### 2.1. Ball-Rolling Dung Beetles

Dung beetles constantly update their position in sunlight by sensing environmental factors such as sunlight or wind direction, a behavior that can be accurately described by the mathematical model described in Equation (1).

(1) $$\begin{array}{c} x_{i}\left(t+1\right)=x_{i}\left(t\right)+\alpha \times k\times x_{i}\left(t-1\right)+b\times △x,\\ △x=\left|x_{i}\left(t\right)-X^{w}\right|. \end{array}$$

*t* denotes the current iteration number, $x_{i}\left(t\right)$ denotes the position of the *i*-th dung beetle at the *t*-th iteration, α denotes whether the dung beetle’s direction deviates or not, and its value is set to 1 or −1 according to the probability, with 1 denoting no deviation and −1 denoting a deviation. k∈(0,0.2] is a deflection coefficient, and *b* denotes a constant that belongs to (0,1), and $X^{w}$ is used to denote the global worst position, and △x is used to simulate the change of light intensity. Rolling dung beetles have a certain probability of encountering an obstacle, when the rolling dung beetle encounters an obstacle and cannot proceed further, the dung beetle acquires a new direction by dancing, and the dancing behavior is defined in Equation (2).

(2) $$x_{i}\left(t+1\right)=x_{i}\left(t\right)+tan\left(\theta \right)\left|x_{i}\left(t\right)-x_{i}\left(t-1\right)\right|.$$

where $\theta \in \left[0,\pi \right]$, the position of the dung beetle will not be updated when the values of θ are π/2 and π.

### 2.2. Breeding Dung Beetles

**Figure 1:**
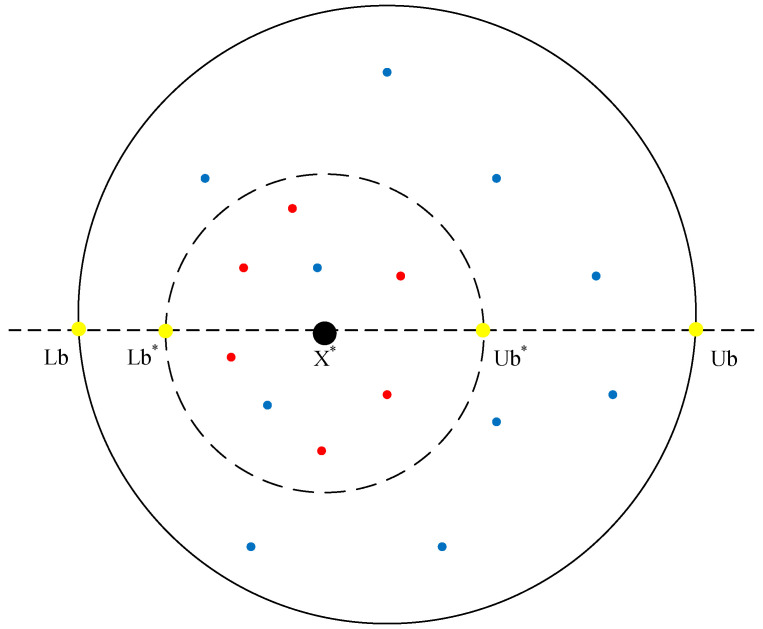
Boundary selection strategy.

In order to provide a safe environment for the offspring, dung beetles will spawn in a safe area, and a safe spawning area is defined as a boundary selection strategy as shown in Equation (3).

(3) $$\begin{array}{c} R=1-t/T_{max},\\ Lb^{*}=max\left(X^{*}\times \left(1-R\right),Lb\right),\\ Ub^{*}=min\left(X^{*}\times \left(1+R\right),Ub\right). \end{array}$$

where *R* denotes the convergence factor, $T_{max}$ denotes the maximum number of iterations, $X^{*}$ denotes the local optimal position, $Lb^{*}$ and $Ub^{*}$ denote the lower and upper boundaries of the spawning area, respectively, and Lb and Ub denote the lower and upper bounds of the objective function. As shown in Figure 1, the outermost large circle represents the upper and lower boundaries of the optimization problem, and the inner circle represents the area where the dung beetles breed. $X^{*}$ is denoted by the black ball, the red dots denote the positions of the breeding balls, the blue dots denote the positions of the rolling dung beetles, and the yellow dots denote the boundaries. When the spawning area is determined, each female dung beetle lays an egg in the area of the spawning area in each iteration.

From Equation (3), it is found that the spawning area follows the value of *R* dynamically, therefore, the location of the laid egg also changes dynamically, and the spawning location is defined in Equation (4).

(4) $$B_{i}\left(t+1\right)=X^{*}+b_{1}\times \left(B_{i}\left(t\right)-Lb^{*}\right)+b_{2}\times \left(B_{i}\left(t\right)-Ub^{*}\right).$$

In this context, $B_{i}\left(t\right)$ denotes the location of the *i*-th breeding ball at the *t*-th iteration, $b_{1}$ and $b_{2}$ denote 1×D independent random vectors, *D* denotes the dimension of the optimization problem, and the symbol ‘×’ means two vectors conduct element-wise multiplication. The position of the breeding ball is strictly limited to the spawning area.

### 2.3. Foraging Dung Beetles

When young dung beetles forage, they also need to establish the best foraging area to guide them to forage, and the foraging area is defined in Equation (5).

(5) $$\begin{array}{c} Lb^{b}=max\left(X^{b}\times \left(1-R\right),Lb\right),\\ Ub^{b}=min\left(X^{b}\times \left(1+R\right),Ub\right). \end{array}$$

where the $Lb^{b}$ and $Ub^{b}$ sub-tables denote the lower and upper bounds of the best foraging area and $X^{b}$ denotes the global best position. The location update of the small dung beetle is defined in Equation (6).

(6) $$x_{i}(t+1)=x_{i}\left(t\right)+C_{1}\times \left(x_{i}\left(t\right)-Lb^{b}\right)+C_{2}\times \left(x_{i}\left(t\right)-Ub^{b}\right).$$

$C_{1}$ is a random number obeying a normal distribution and $C_{2}$ denotes a random vector belonging to (0,1).

### 2.4. Stealing Dung Beetles

The stealing behavior denotes stealing dung balls from other dung beetles. During the iterative process, the location information update strategy of the thief dung beetle is defined in Equation (7).

(7) $$x_{i}(t+1)=X^{b}+S\times g\times \left(\left|x_{i}\left(t\right)-X^{*}\right|+\left|x_{i}\left(t\right)-X^{b}\right|\right).$$

where *S* denotes a constant and *g* is a random vector of size obeying a normal distribution.

### 2.5. The DBO Algorithm Implementation Steps

**Figure 2:**
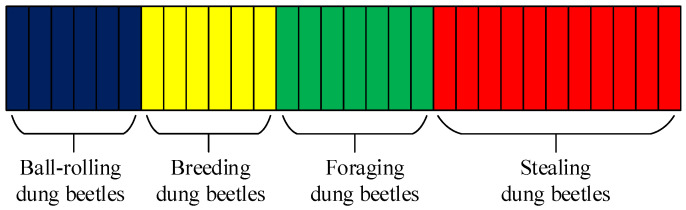
Species distribution.

The distribution of the population in DBO is shown in Figure 2, where the number of matrices indicates the number of dung beetles, and the blue, yellow, green, and red matrices represent ball-rolling dung beetles, breeding dung beetles, foraging dung beetles, and stealing dung beetles, respectively. The overall pseudo-code of the DBO algorithm is shown in Algorithm 1.
**Algorithm 1:** The framework of the DBO algorithm.
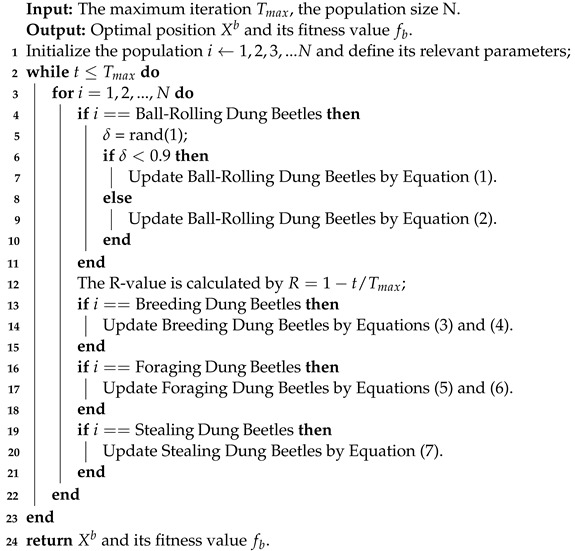


## 3. Multi-Strategy Improved Dung Beetle Optimization Algorithm (MDBO)

The basic characteristics of the dung beetle optimization algorithm can be derived from its principle. The ball-rolling behavior enhances the global search ability of the algorithm across all phases, while reproduction and foraging behaviors facilitate exploration around the optimal position of the individual. With each iteration, the dynamic boundary and range of search decrease gradually. The stealing behavior entails a dynamic localized search near the optimal individual. Despite the simplicity of the DBO algorithm and its successful application in certain engineering design problems, it exhibits several drawbacks. Striking a balance between global exploration and local exploitation poses challenges, and algorithms are prone to falling into local optima [27]. To rectify these issues, this study proposes enhancements in the ensuing sections.

### 3.1. Latin Hypercube Sampling to Initialize Populations

The DBO algorithm usually relies on a stochastic initialization strategy to generate the initial population when solving complex optimization problems. This randomization helps to explore different regions of the solution space, thus increasing the chance of finding a globally optimal solution. However, random initialization also has an obvious drawback: it cannot ensure the uniform distribution of the population in the solution space. Especially in high-dimensional search spaces, it requires a large number of points to obtain a good distribution, and these points may be close to each other or even overlap [28]. This may result in the population being too concentrated in some regions and too sparse in others. This uneven distribution is very detrimental to the early convergence of the algorithm.

To address this issue, this study introduces an initialization method called Latin hypercube sampling (LHS) [29,30]. The fundamental concept of LHS involves partitioning the sample space into multiple uniform and non-overlapping subspaces and selecting a single data point from each subspace as a sampling point. This approach guarantees a uniform distribution of sample points across the defined domain, thereby mitigating the risk of over-concentration or sparse distribution of agents. Mathematically, the generated sample is represented using Equation (8).

(8) $$x_{i}=\frac{1}{n}r+\frac{i-1}{n}.$$

where *r* is a uniform random number in (0,1), $x_{i}$ is the sample in the ith interval, and *n* is the total number of samples. When the total number of samples is 10, the sample $x_{1}=\frac{1}{10}r+\frac{0}{10}$ in the first interval has a range of [0,0.1], and similarly the sample in the second interval has a range of [0.1,0.2], and so on to obtain all the sampling points of all LHSs.

**Figure 3:**
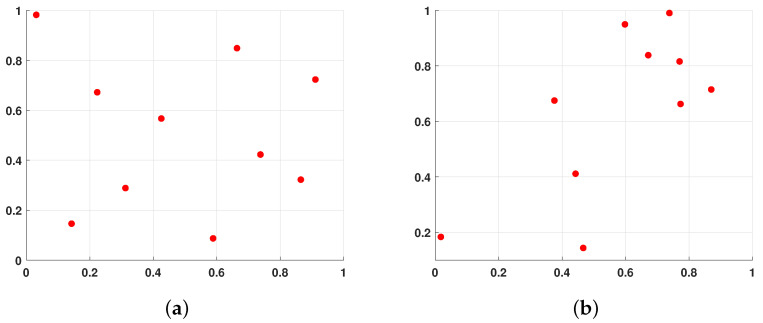
Comparison of 10 point sets generated by LHS and 10 randomly generated point sets. Where (**a**) denotes the 10 point sets generated by LHS and (**b**) denotes the 10 randomly generated point sets.

Compared to random or stratified sampling methods, Latin hypercube sampling (LHS) exhibits stronger spatial filling capability and convergence characteristics [31]. This attribute has led to its widespread application in the initialization of populations in intelligent algorithms. Figure 3 illustrates a two-dimensional comparison between the distributions of 10 randomly generated populations and populations generated using LHS. It is evident from the figure that the population distribution generated by LHS is more uniform, with no overlapping individuals. Therefore, this method can generate higher-quality initial populations, laying a better foundation for subsequent algorithm optimization.

As a metaheuristic algorithm based on swarm intelligence, the dung beetle optimization algorithm is mathematically modeled in the same way at initialization as other algorithms as shown in Equation (9). The set of points acquired through LHS can often be mapped to the solution space of the objective function using an equation similar to the one depicted in Equation (10).

(9) $$X=\left[\begin{array}{c} X_{1}\\ \vdots \\ X_{i}\\ \vdots \\ X_{N} \end{array}\right]={\left[\begin{array}{ccccc} x_{1,1} & \cdots & x_{1,i} & \cdots & x_{1,D}\\ \vdots & \ddots & \vdots & \ddots & \vdots \\ x_{i,1} & \cdots & x_{i,i} & \cdots & x_{i,D}\\ \vdots & \ddots & \vdots & \ddots & \vdots \\ x_{N,1} & \cdots & x_{N,i} & \cdots & x_{N,D} \end{array}\right]}_{N\times D}$$

(10) $$X_{i}=lb+\left(ub-lb\right)\times LHS_{i}.$$

Here, *X* is the population matrix, $X_{i}$ is the *i*th DBO member (candidate solution), *N* is the number of dung beetles, *D* is the number of decision variables, lb and ub represent the upper and lower bounds of the problem to be optimized, $LHS_{i}$ denotes the *i*th vector obtained using Latin Hypercube Sampling.

### 3.2. Mean Differential Variation

Throughout the iterative process, as the population gradually converges towards optimal individuals, there is a tendency for decreased population diversity. To prevent premature convergence of the algorithm caused by a reduction in population diversity throughout the iteration process, this paper introduces the mean differential variation [32]. Depending on the stage of the iteration, this method can be categorized into two variants, denoted as DE/mean-current/1 and DE/mean-current-best/1 respectively. Both variants initially select two individuals, $X_{r1}$ and $X_{r2}$, randomly from the current population, and calculate two new vectors, $X_{c1}$ and $X_{c2}$, according to Equation (11).

(11) $$\begin{array}{c} X_{c1}=\frac{X_{r1}+X_{r2}}{2},\\ X_{c2}=\frac{X_{r1}+X^{b}}{2}. \end{array}$$

The first variation strategy, which proceeds according to Equation (12), is unique in that it employs two fundamental vectors external to the current population. This strategy not only helps to escape the problem of population stagnation but also effectively maintains the diversity of the population, thus promoting the exploration capability of the algorithm. Consequently, the algorithm is able to search in a wider solution space, thereby augmenting the likelihood of discovering a globally optimal solution.

(12) $$X_{i}=X_{c1}+F\left(X_{c1}-X_{i}\right)+F\left(X_{c2}-X_{i}\right).$$

The second variation strategy is executed based on Equation (13), where the generation of new vectors incorporates information about the global optimal solution. This improvement allows the algorithm to perform a more intensive search in the vicinity of the optimal solution, thus finely exploring small variations in the solution space. In this way, the algorithm is able to approximate the global optimal solution more accurately, improving the accuracy and efficiency of the solution.

(13) $$X_{i}=X^{b}+F\left(X_{c1}-X_{i}\right)+F\left(X_{c2}-X_{i}\right).$$

In Equations (12) and (13), $X^{b}$ represents the current best individual, $X_{i}$ denotes the individual currently undergoing mutation, and *F* is the scaling factor. In the first type of mutation, F=0.25, while in the second type of mutation, F=(1−2×rand(1))×0.5. Both types of mutations are executed in a cooperative manner. In the first two-thirds of the iterations, the first type of mutation is exclusively performed as it provides good search and exploitation capabilities. In the last one-third of the iterations, the second type of mutation is executed to conduct a more intensive search. Overall, as in Equation (14), we have the following:

(14) $$\left\{\begin{array}{cc} \left\{\begin{array}{c} F=0.25\\ X_{i}=X_{c1}+F\left(X_{c1}-X_{i}\right)+F\left(X_{c2}-X_{i}\right) \end{array}\right. & t<T_{\max}*\frac{2}{3},\\ \left\{\begin{array}{c} F=(1-2\times \mathrm{rand}(1))\times 0.5\\ X_{i}=X^{b}+F\left(X_{c1}-X_{i}\right)+F\left(X_{c2}-X_{i}\right) \end{array}\right. & otherwise. \end{array}\right.$$

This strategy of searching near individuals in the early stages and exploring near the global optimum in the later stages effectively helps the algorithm escape local optima, thereby enhancing the algorithm’s global search capability and convergence speed.

### 3.3. Fusion Lens Imaging Backward Learning and Dimension-by-Dimension Optimization

**Figure 4:**
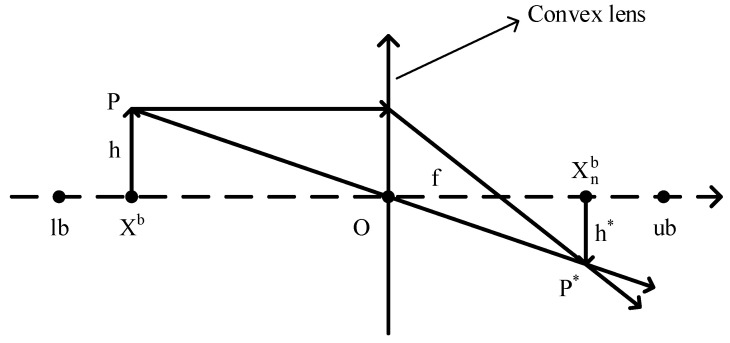
Lens imaging reverse learning.

The position of the current best individual is particularly important, but in the basic dung beetle optimization (DBO) algorithm, the information contained in the current best individual is not fully utilized, leading to a lack of exploitation of the best individual. Therefore, this paper introduces the lens imaging reverse learning strategy [33,34] to perturb the best individual to help the algorithm escape local optima. The idea is to generate a reverse position based on the current coordinates to expand the search range, which can effectively avoid local optima and broaden the search scope of the algorithm. The principle of the lens imaging reverse learning strategy is depicted in Figure 4.

Suppose within a certain space, the global optimal position $X^{b}$ is obtained by projecting an individual *P* with a height of *h* onto the x-axis. Here, lb and ub represent the lower and upper limits of the coordinate axis. Placing a convex lens with a focal length *f* at the origin *O*, a point $P^{*}$ with a height $h^{*}$ can be obtained through the convex lens. At this point, the projection $X_{n}^{b}$ of $P^{*}$ on the x-axis is the reverse solution. According to the principle of lens imaging, Equation (15) can be derived.

(15) $$\frac{\frac{lb+ub}{2}-X^{b}}{X_{n}^{b}-\frac{lb+ub}{2}}=\frac{h}{h^{*}}.$$

Let $\frac{h}{h^{*}}=k$, and by transformation, we obtain Equation (16).

(16) $$X_{n}^{b}=\frac{ub+lb}{2}+\frac{ub+lb}{2\cdot k}-\frac{X^{b}}{k}.$$

By adjusting the value of *k* in the lens imaging reverse learning, the dynamic reverse solution can be obtained. A smaller *k* produces a larger range of inverse solutions, while a larger *k* can produce a smaller inverse. This paper introduces an adaptive *k* as Equation (17). As the number of iterations increases, the value of *k* will grow from small to large, to meet the characteristics of a large-scale search in the early stage and a fine search in the late stage.

(17) $$k={\left(1+{\left(\frac{t}{T_{max}}\right)}^{0.5}\right)}^{10}.$$

In dung beetle optimization (DBO), each agent represents a potential solution. When updating each agent, updates are made across all dimensions, overlooking the changes in dimensions within each agent. Suppose a dimension within an agent moves towards a better solution, but degradation in other dimensions leads to a decrease in the overall solution quality, resulting in the abandonment of that solution. This would waste evaluation efforts and deteriorate convergence speed [35]. Based on a greedy per-dimension update strategy, the evolutionary dimension of solutions will not be overlooked due to degradation in other dimensions, allowing any update value that can improve the solution to be accepted. Ensuring that the algorithm can utilize evolutionary information from individual dimensions for better local search, thereby obtaining higher-quality solutions and improving the convergence speed [36].

In this paper, a strategy combining lens imaging reverse learning and dimension-by-dimension optimization. The core idea of this strategy lies in updating the best value obtained through lens imaging reverse learning in a per-dimension manner, combined with greedy rules to optimize the solution. Specifically, initially, a mutation operation is applied to the best individual $X^{b}$ as shown in Equation (16), resulting in a mutated individual $X_{n}^{b}$. Subsequently, the fitness values of $X^{b}$ and $X_{n}^{b}$ are compared, and the individual with better fitness is chosen as the benchmark position. Then, all dimensions of another position are used to replace the corresponding dimensions of the benchmark position one by one. In the process of per-dimension replacement, a greedy rule is adopted: If the overall fitness value improves after replacing a dimension, the replaced value of that dimension is retained; otherwise, the benchmark position remains unchanged. Through such per-dimension optimization, the structure of the solution can be finely adjusted, further enhancing the quality of the solution. Finally, the reference position after dimension replacement becomes the new $X^{b}$ of the next generation. This process integrates the idea of lens imaging reverse learning and dimensional optimization, aiming to approach the global optimal solution gradually through continuous iteration and optimization. The complete algorithm flow is shown in Algorithm 2.
**Algorithm 2:** Fusion lens imaging backward learning and dimension-by-dimension optimization strategies.
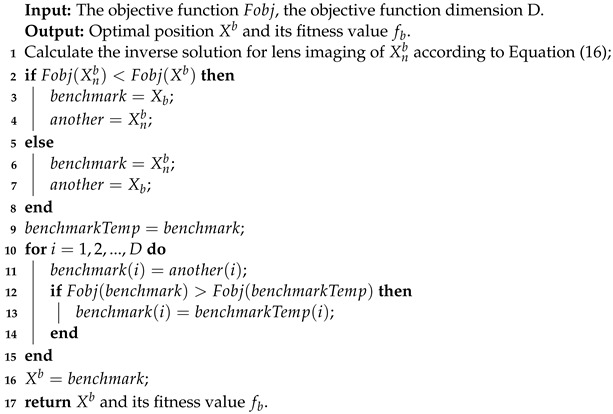


### 3.4. Complexity Analysis of MDBO

Assume that N represents the number of populations, D represents the dimension of the optimization problem, and T represents the maximum number of iterations, DBO exhibits an initialization phase complexity of $t_{1}$ = O(N*D) and an iterative process complexity of $t_{2}$ = O(T*N*D), resulting in a total complexity of $t_{1}$ + $t_{2}$ = O(T*N*D). For MDBO, the complexity of initializing the population using Latin hypercube is $t_{3}$ = O(N*D), the average differential variance complexity is $t_{4}$ = O(N*T), the complexity of fusing lens imaging reverse learning and dimension-by-dimension optimization is $t_{5}$ = O(T*D), and the complexity of the iterative process is the same as that of DBO as $t_{2}$. Hence, the complexity of MDBO is $t_{2}$ + $t_{3}$ + $t_{4}$ + $t_{5}$ = O(T*N*D), equivalent to DBO, and its performance does not depend on the higher complexity.

### 3.5. The MDBO Algorithm Implementation Steps

**Figure 5:**
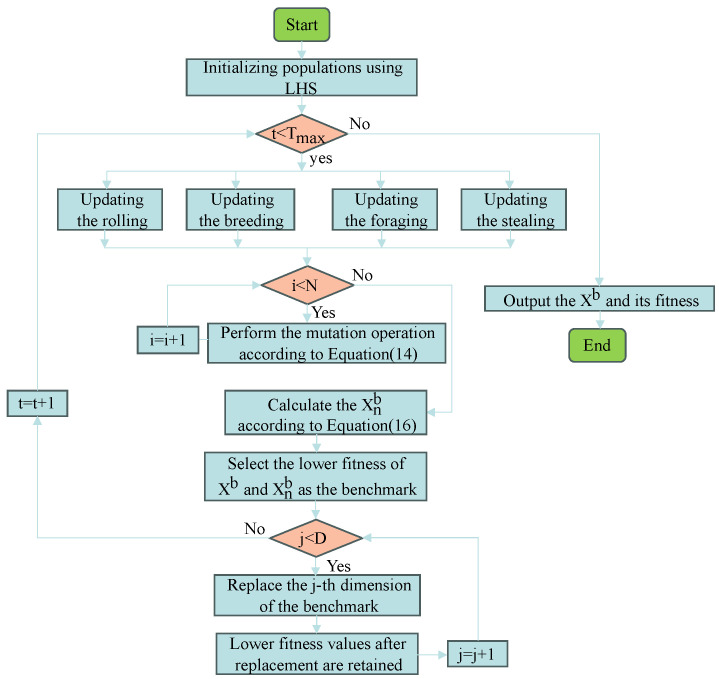
The flowchart of MDBO.

The basic framework of the MDBO algorithm is outlined in Algorithm 3. To provide a clear visualization of the process, Figure 5 illustrates the flowchart of MDBO. This algorithm aims to enhance search efficiency and convergence speed during optimization by employing a combination of multiple strategies. Specifically, the MDBO algorithm utilizes Latin hypercube sampling for improved population initialization and introduces a novel differential variation strategy called “Mean Differential Variation” to enhance its ability to evade local optima. Moreover, applying lens imaging reverse learning to the current optimal solution to expand the algorithm’s search space, and combining it with a dimension-by-dimension optimization strategy to improve the quality of the solution.
**Algorithm 3:** The framework of the MDBO algorithm
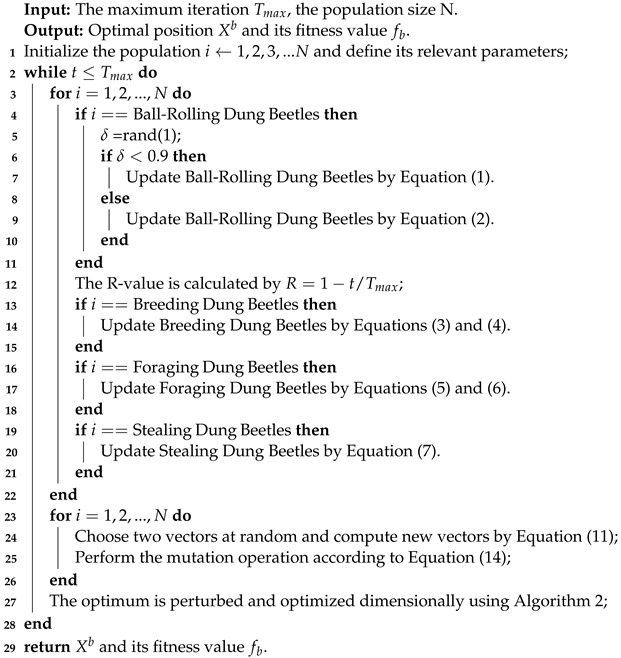


## 4. Experimental Results and Discussions

In order to evaluate the performance of the improved algorithm comprehensively, this paper selects two sets of benchmark functions: CEC2017 [37] and CEC2020 [38]. The details of the benchmark functions are shown in Table 1. In CEC2017, the original F2 function has been excluded due to loss of testing capability, thus leaving 29 single-objective benchmark functions for testing. Among these, F1 and F2 are single-peaked functions with only one global minimum, F3–F9 are simple multi-modal functions with local minima, F10–F19 are mixed functions containing three or more CEC2017 benchmark functions after rotation or displacement, and F20–F29 are composite functions formed by at least three mixed functions or CEC2017 benchmark functions after rotation and displacement. CEC2020 consists of one composite single-peaked function F1, three multi-peaked functions F2–F4 after rotation and displacement, three mixed functions F5–F7, and three composite functions F8–F10.

The comparison algorithms encompass DBO [25], WOA [20], GWO [19], SCA [14], SSA [22], HHO [21]. To ensure the fairness of the experiments, the initial population size for all algorithms is set to 30, and the maximum number of iterations is set to 500. To eliminate the influence of randomness in the experiments, each algorithm is independently executed 30 times to statistically analyze its results. MATLAB R2020b is utilized for software implementation.

### 4.1. CEC2017 Test Function Results and Analysis

#### 4.1.1. Analysis of CEC2017 Statistical Results

The statistical outcomes for the CEC2017 test function in 30 and 100 dimensions were meticulously documented. These include the minimum (min), mean, and standard deviation (std) of each algorithm’s independent execution conducted 30 times. The best average result for each test function is accentuated in bold font. The last row “Total” indicates the number of times each algorithm achieved the best result among all test functions. The statistical results for 30 and 100 dimensions are presented in Table 2 and Table 3, respectively.

From Table 2 and Table 3, the comprehensive analysis reveals that, overall, for the 30-dimensional case, MDBO obtained the optimal solution in 21 out of 29 test functions. For the remaining 8 test functions, MDBO achieved the second-best result, while GWO obtained 2 optimal values and SSA obtained 6 optimal values. However, in the case of 100 dimensions, MDBO only attained the best solution in 15 out of 29 test functions, yielding suboptimal outcomes in the remaining 14 test functions. A detailed examination reveals the following:

- MDBO did not achieve the best performance among all algorithms on the unimodal function F1, whether in the 30-dimensional or 100-dimensional case. It demonstrated superior performance compared to other algorithms but fell short of SSA. Notably, MDBO excelled in the unimodal function F2, outperforming all algorithms in both 30 and 100 dimensions.
- In the simple multimodal problems F3–F9, MDBO achieved the best average fitness value in 6 out of 7 test functions in the 30-dimensional scenario, except for F5, where it trailed slightly behind GWO. However, in the 100-dimensional scenario, MDBO exhibited weaker performance compared to GWO on functions F4, F5, F6, and F7, and weaker than SSA on functions F3, F8, and F9. Nevertheless, an improvement was observed in all benchmark functions compared to the basic DBO.
- For the hybrid functions F10–F19, in the 30-dimensional scenario, MDBO obtained the minimum values on all 7 test functions compared to the other algorithms. It ranked second after SSA in functions F11, F12, and F18. In the 100-dimensional scenario, MDBO secured minimum values in 7 out of 10 test functions, excluding F11, F13, and F17.
- In the case of composite functions F20–F29, when the dimension is 30, MDBO only did not achieve the best results on F24, F27, and F28 but secured the top position in the remaining 7 functions. When the dimension is 100, MDBO exhibited weaker performance compared to SSA in functions F21, F24, and F27 but obtained the best results in the remaining 7 functions.

**Figure 6:**
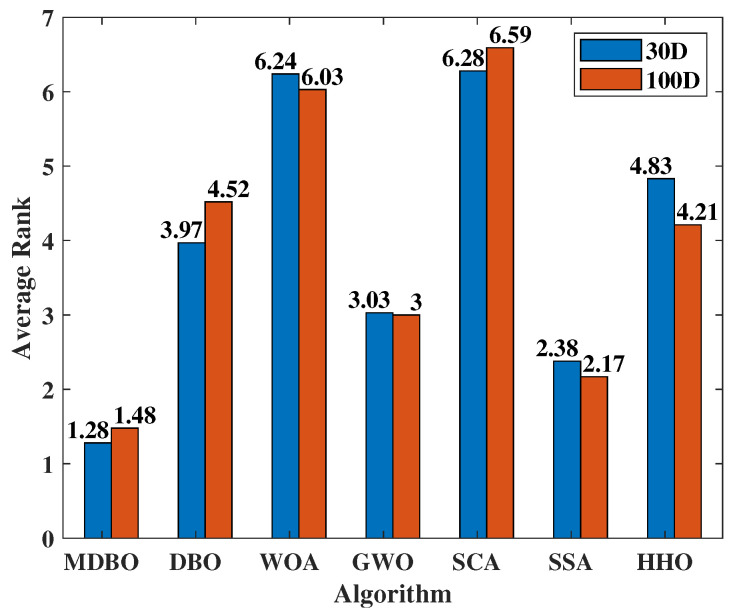
CEC2017 average rank.

To comprehensively evaluate the performance of all algorithms, this study conducted Friedman tests on the average results of 30 independent optimization runs for 30 test functions for each algorithm. The average rankings of all algorithms on the test functions were calculated, where a lower average ranking indicates better algorithm performance. The Friedman test results for dimensions 30 and 100 are shown in Figure 6. From the results, it is evident that the average rankings for dimensions 30 and 100 maintain similar trends, with MDBO achieving the lowest average ranking, followed by SSA, GWO, DBO, HHO, WOA, and SCA, respectively. This suggests that, compared to other algorithms, MDBO generally exhibits superior performance.

#### 4.1.2. CEC2017 Convergence Curve Analysis

**Figure 7:**
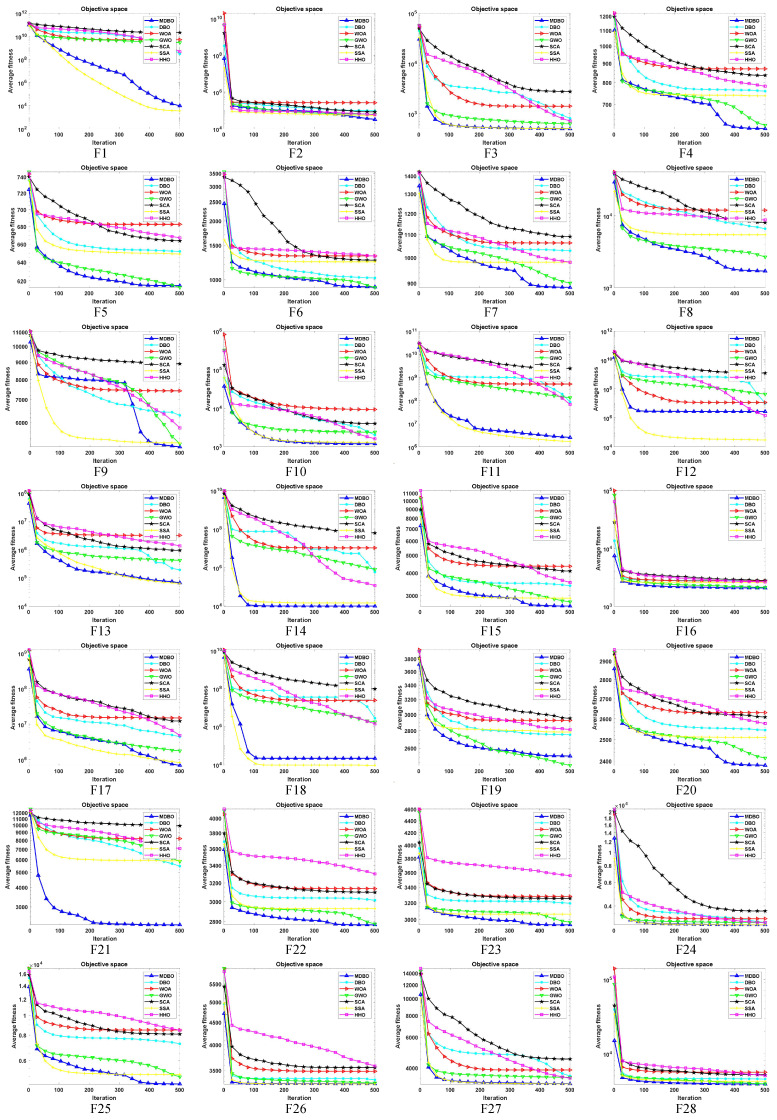
The CEC2017 iteration curve when the dimension is 30.

**Figure 8:**
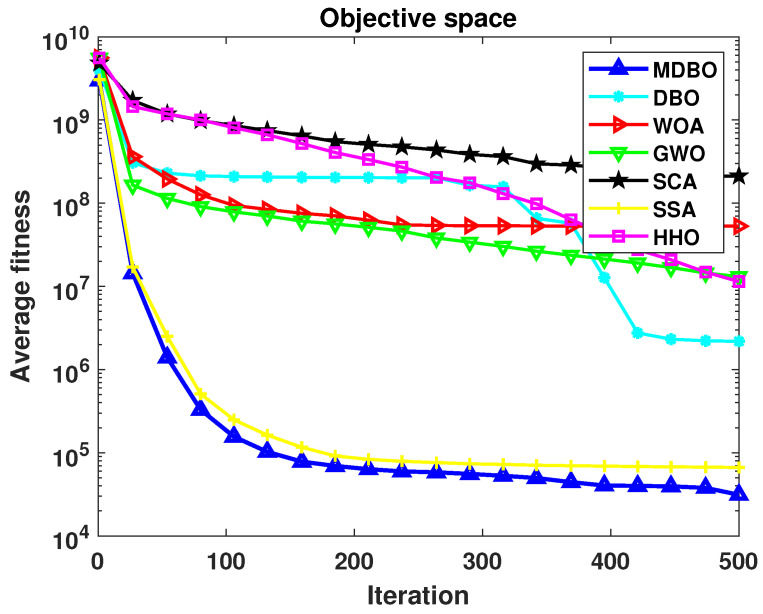
F29 convergence curve.

In order to assess both the accuracy and convergence speed of the algorithms, convergence curves were plotted for MDBO and other algorithms at dimension 30, as illustrated in Figure 7. It is worth noting that in each subplot, the horizontal axis represents the number of iterations, while the vertical axis represents the average convergence curve over 30 runs. From the figure, the following can be observed:

- For the unimodal problem F1, initially, the convergence speed of MDBO was slower than SSA. However, after approximately two-thirds of the iterations, its convergence speed accelerated and gradually caught up with SSA, achieving results close to SSA. As for unimodal problem F2, the convergence speed of MDBO was comparable to other comparative algorithms. However, owing to its superior exploration capability, MDBO converged to a better solution.
- For the simple multimodal functions F3, MDBO, and SSA exhibited comparable convergence speed and accuracy, outperforming all other comparative algorithms. Concerning F4, F7, F7, and F9, initially, only the convergence speeds of GWO and SSA were similar to MDBO. However, after around two-thirds of the iterations, the convergence speeds of SSA and GWO slowed down, while the convergence speed of MDBO accelerated, rapidly converging to better positions. Regarding F5 and F6, the convergence speed of MDBO was on par with GWO and superior to other algorithms.
- In the case of hybrid functions F10–F19, MDBO demonstrated decent convergence speed, particularly excelling in F15 and F19, maintaining a leading position consistently. Concerning F10, F11, F13, F14, F17, and F18, MDBO exhibited similar convergence speed and accuracy to SSA. Regarding F12, MDBO’s performance was inferior to SSA but significantly outperformed other comparative algorithms, showing a substantial improvement over DBO. As for F16, the results obtained by all algorithms were similar, with minor differences.
- In the case of composite functions F20, F21, F22, F23, and F25, MDBO consistently demonstrated the fastest convergence speed and accuracy, outperforming all other comparative algorithms, especially evident in F21, where it significantly surpassed other algorithms. Regarding F24, F26, F27, and F28, MDBO’s performance was comparable to other algorithms, slightly superior in certain benchmark functions. Concerning F29, the results are shown in Figure 8, which can be found that the convergence speed of SSA and MDBO was similar, but MDBO had a slight edge.

### 4.2. CEC2020 Test Function Results and Analysis

#### 4.2.1. Analysis of CEC2020 Statistical Results

The experimental statistical findings for the CEC2020 test function with a dimension of 20 are depicted in Table 4. This table meticulously records the minimum (min), mean, and standard deviation (std) values resulting from 30 independent runs for each algorithm. Notably, the best average result among all algorithms is marked in bold. Furthermore, the concluding row of the table provides a tally of occurrences wherein each algorithm attained the optimal value across all test functions. From Table 4, it becomes apparent that MDBO outperformed its counterparts in nine test functions, with only a marginal deviation observed in comparison to GWO in the F3 test function.

**Figure 9:**
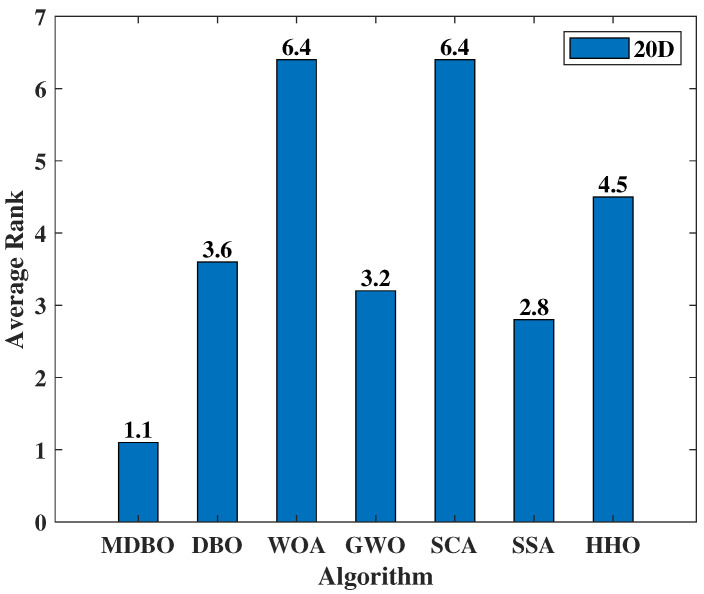
CEC2020 average rank.

In employing Friedman’s test, an assessment of the average rank for each algorithm across all test function outcomes was undertaken. As delineated in Figure 9, a discernible tendency is found: MDBO achieves the lowest rank, followed by SSA, GWO, DBO, HHO, WOA, and SCA. This unequivocally underscores the pronounced superiority of MDBO over its algorithmic counterparts.

#### 4.2.2. CEC2020 Convergence Curve Analysis

**Figure 10:**
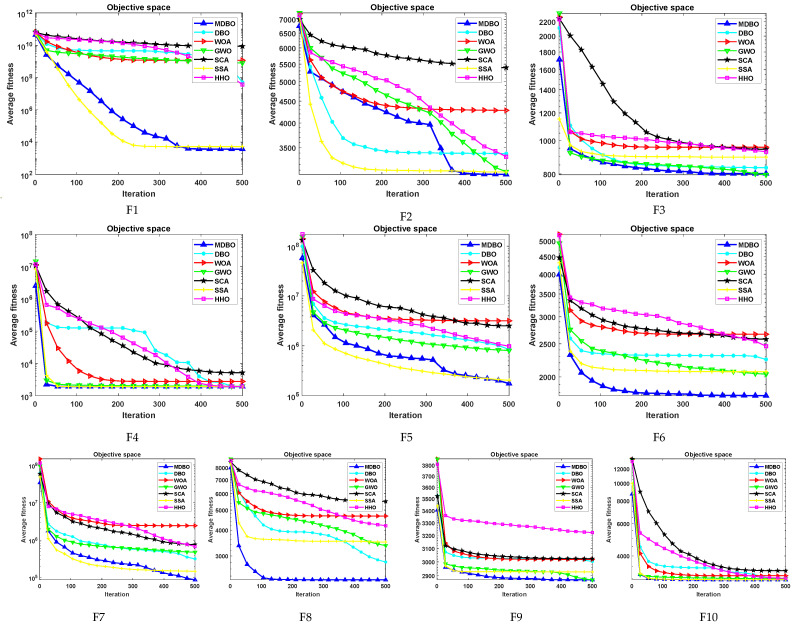
CEC2020 iteration curve when the dimension is 20.

Similarly, the average convergence curves for CEC2020 in 20 dimensions were plotted as shown in Figure 10. It can be observed that in the unimodal function F1, SSA initially exhibited faster convergence compared to MDBO. However, as iterations progressed, SSA became trapped in local optima, while MDBO demonstrated superior exploration capability, eventually discovering better solutions. In F2, MDBO initially exhibits slower convergence compared to DBO and SSA. Nevertheless, as DBO and SSA fall into local optima, MDBO maintains a decent convergence rate. In F6, F7, and F8, MDBO significantly outperforms other comparative algorithms in terms of convergence speed, precision, and stability. Moreover, MDBO demonstrates varying degrees of superiority in the remaining test functions. This robustly validates the effectiveness of MDBO in addressing complex optimization problems.

### 4.3. Wilcoxon Rank Sum Test

The Wilcoxon rank-sum test [39,40] is a non-parametric statistical test used to further determine whether the differences between the improved algorithm and the comparative algorithms are significant. In this study, the results of running the six comparative algorithms and MDBO 30 times were used as samples. The Wilcoxon rank-sum test was applied at a significance level of 0.05. When the test result’s *p*-value is less than 0.05, it indicates a significant difference between the two compared algorithms; otherwise, it suggests that the results of the two algorithms are comparable.

*p*-values of the Wilcoxon rank-sum test for CEC2017 at dimensions 30 and 100 are displayed in Table 5 and Table 6, respectively. The *p*-values of the Wilcoxon rank-sum test for CEC2020 at dimension 20 are shown in Table 7. Values with *p*-values greater than 0.05 are highlighted in bold. The last row of each table summarizes the number of times all comparative algorithms had *p*-values less than 0.05 across all test functions.

Based on the results in Table 5, it is evident that at a dimension of 30 in CEC2017, MDBO exhibits significant disparities when compared to both WOA and SCA across all test functions. In contrast, when juxtaposed with DBO and HHO, MDBO shows significant differences in all 28 test functions. Moreover, in comparison with GWO and SSA, MDBO demonstrates significant disparities in 18 and 21 test functions, respectively. Further scrutiny of Table 6 reveals that as the dimension increases to 100, MDBO exhibits significant differences compared to DBO, WOA, and SCA across all functions. When compared to GWO, SSA, and HHO, only a minority of functions show similar results, with significant differences apparent in the majority of cases.

Furthermore, according to Table 7, among the ten benchmark functions of CEC2020, it is evident that MDBO exhibits comparable performance with GWO in functions F7 and F9, whereas, in the remaining test functions, MDBO demonstrates a significant advantage. Conversely, when compared to SSA, significant differences are observed in only 5 test functions. However, when compared to DBO, WOA, SCA, and HHO, MDBO consistently demonstrates absolute superiority across all test functions.

### 4.4. Summary of Experiments

Upon scrutinizing the statistical results and convergence curves derived from CEC2017 at 30 dimensions, it becomes evident that MDBO exhibits superior optimization capabilities characterized by enhanced seeking ability, greater stability, accelerated convergence speed, and heightened convergence accuracy compared to its algorithmic counterparts. This trend persists even when the dimensionality is increased to 100, as MDBO continues to demonstrate commendable performance in tackling high-dimensional optimization challenges. To further validate the efficacy of MDBO in addressing complex problem landscapes, additional experimentation was conducted using CEC2020, which reaffirmed MDBO’s consistent and robust performance in handling intricate optimization scenarios, underscoring its adaptability and reliability in real-world applications.

In order to verify the difference between MDBO and other algorithms, the *p*-values of CEC2017 at 30 dimensions, 100 dimensions, and CEC2020 at 20 dimensions were calculated using the Wilcoxon rank sum test. The results show that MDBO is significantly different from other algorithms and has obvious advantages.

Through performance testing of the MDBO algorithm from multiple aspects, it is evident that MDBO exhibits noteworthy competitiveness in terms of convergence speed, accuracy, stability, and robustness when juxtaposed against contemporary algorithms. Moreover, its performance remains steadfast even amidst the complexities of high-dimensional optimization challenges, affirming the efficacy and relevance of MDBO in modern optimization contexts.

## 5. Engineering Application Design Issues

To further validate the reliability of MDBO in practical engineering applications, three typical engineering design problems are employed to assess its optimization performance across various practical scenarios. These problems include extension/compression spring design problems [41], reducer design problems [42], and welded beam design problems [43].

The engineering design optimization problem is classified as a constrained optimization problem involving variables, necessitating dealing with constraints [44]. Three primary methods are commonly employed for constraint processing: The penalty function method, feasibility rule, and multi-objective method. In this study, the external penalty function method is adopted, whereby constraints are transformed into penalty functions, thus integrating them with the objective function. This integration results in a new objective function, defined in Equation (18).

(18) $$F\left(\vec{x}\right)=f\left(\vec{x}\right)+w\cdot {\left(\sum_{i=1}^{m}\left(max\left(0,g_{i}\left(\vec{x}\right)\right)\right)\right)}^{2},$$

$F(\vec{x})$ represents the fitness function value, while $f(\vec{x})$ and $g_{i}\left(\vec{x}\right)$ represent the objective function value and the constraint function, respectively. *w* is the penalty parameter of the penalty function, which is set to 10e100 in this article. *w* makes the violation of constraints in the optimization process will be punished, so as to find the optimal solution satisfying the constraints.

In the experimental comparison of algorithms for design problems in engineering applications, the comparison algorithms are DBO [25], WOA [20], GWO [19], SCA [14], SSA [22], HHO [21], and for all algorithms, the population size is set to 30 and the maximum number of iterations is 500. In practical engineering scenarios, the reliability of optimization algorithms is crucial. While high average trial run values may initially indicate promising performance, large standard deviations can signal instability and unreliability, particularly in computationally expensive real-world problems where multiple trial runs may not be feasible due to limited computational resources. Therefore, to ensure robustness and reliability, this study conducts 30 independent runs of each algorithm and computes both the mean and standard deviation of their performance metrics. This approach provides a comprehensive evaluation, accounting for both average performance and stability, essential for assessing algorithm suitability in real-world engineering applications.

### 5.1. Extension/Compression Spring Design Issues

**Figure 11:**
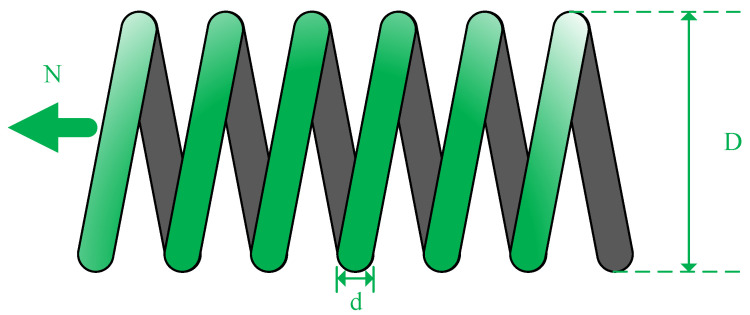
Extension/compression spring design issues.

The extension/compression spring design problem, illustrated in Figure 11, seeks to minimize spring weight by optimizing parameters such as wire diameter (*d*), average coil diameter (*D*), and the number of active coils (*N*). The optimization variables are defined by Equation (19), while the objective function is abstracted as in Equation (20). Constraints are formulated in Equation (21), and upper and lower boundaries are set by Equation (22). This problem endeavors to identify the optimal parameter combination to achieve desired performance while simultaneously minimizing spring weight, thereby facilitating efficient and lightweight spring design for diverse applications.

Consider:

(19) $$\vec{x}=\left[x_{1},x_{2},x_{3}\right]=\left[d,D,N\right],$$

Minimize:

(20) $$f\left(\vec{x}\right)=\left(x_{3}+2\right)x_{2}x_{1}^{2},$$

Subject to:

(21) $$\left.\begin{array}{c} \begin{array}{c} g_{1}\left(\vec{x}\right)=1-\frac{x_{2}^{3}x_{3}}{71785x_{1}^{4}}\leq 0\\ g_{2}\left(\vec{x}\right)=\frac{4x_{2}^{2}-x_{1}x_{2}}{12566(x_{2}x_{1}^{3}-x_{1}^{4})}+\frac{1}{5108x_{1}^{2}}\leq 0\\ g_{3}\left(\vec{x}\right)=1-\frac{140.45x_{1}}{x_{2}^{2}x_{3}}\leq 0\\ g_{4}\left(\vec{x}\right)=\frac{x_{1}+x_{2}}{1.5}-1\leq 0 \end{array} \end{array}\right\}$$

Parameters range:

(22) $$0.05\leq x_{1}\leq 2,0.25\leq x_{2}\leq 1.3,2\leq x_{3}\leq 15.$$

The experiment counted the average and standard deviation 30 times solving the results of each algorithm in this problem, and randomly selected the optimal results and optimal parameters of a certain time to show the results, the results are shown in Table 8. It is evident that MDBO achieves the lowest manufacturing cost in solving this problem. Furthermore, the consistency of this result is supported by the mean and standard deviation of the outcomes, indicating its stability and reliability.

### 5.2. Reducer Design Issues

**Figure 12:**
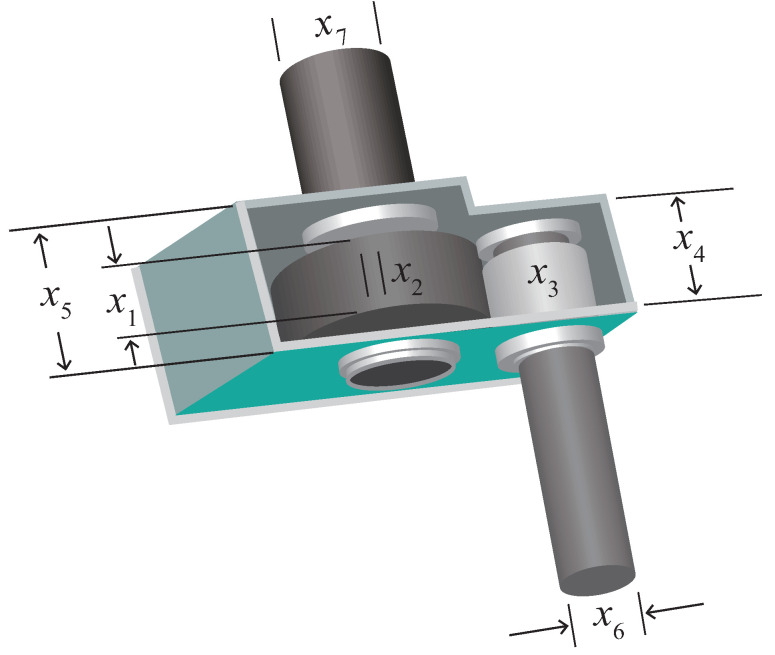
Reducer design issues.

The schematic diagram of the speed reducer design problem is depicted in Figure 12. The problem involves seven design variables, which are end face width ($x_{1}$), number of tooth modules ($x_{2}$), number of teeth in the pinion ($x_{3}$), length of the first shaft between the bearings ($x_{4}$), length of the second shaft between the bearings ($x_{5}$), diameter of the first shaft ($x_{6}$), and diameter of the second shaft ($x_{7}$). The objective of the problem is to minimize the total weight of the gearbox by optimizing seven variables. The objective function is represented by Equation (23), while the constraints are described by Equation (24). The upper and lower bounds for each variable are defined by Equation (25).

Minimize:

(23) $$\begin{array}{c} f\left(\vec{x}\right)=0.7854x_{1}x_{2}^{2}\left(3.3333x_{3}^{2}+14.9334x_{3}-43.0934\right)-1.508x_{1}\left(x_{6}^{2}+x_{7}^{2}\right)+7.4777\left(x_{6}^{3}+x_{7}^{3}\right) \end{array}$$

Subject to:

(24) $$\left.\begin{array}{c} \begin{array}{c} g_{1}\left(\vec{x}\right)=\frac{27}{x_{1}x_{2}^{2}x_{3}}-1\leq 0,\\ g_{2}\left(\vec{x}\right)=\frac{397.5}{x_{1}x_{2}^{2}x_{3}^{2}}-1\leq 0,\\ g_{3}\left(\vec{x}\right)=\frac{1.93x_{4}^{3}}{x_{2}x_{3}x_{6}^{4}}-1\leq 0,\\ g_{4}\left(\vec{x}\right)=\frac{1.93x_{5}^{3}}{x_{2}x_{3}x_{7}^{4}}-1\leq 0,\\ g_{5}\left(\vec{x}\right)=\frac{\sqrt{{\left(\frac{745x_{4}}{x_{2}x_{3}}\right)}^{2}+16.9\times 10^{6}}}{110.0x_{6}^{3}}-1\leq 0,\\ g_{6}\left(\vec{x}\right)=\frac{\sqrt{{\left(\frac{745x_{4}}{x_{2}x_{3}}\right)}^{2}+157.5\times 10^{6}}}{85.0x_{6}^{3}}-1\leq 0,\\ g_{7}\left(\vec{x}\right)=\frac{x_{2}x_{3}}{40}-1\leq 0,\\ g_{8}\left(\vec{x}\right)=\frac{5x_{2}}{x_{1}}-1\leq 0,\\ g_{9}\left(\vec{x}\right)=\frac{x_{1}}{12x_{2}}-1\leq 0,\\ g_{10}\left(\vec{x}\right)=\frac{1.5x_{6}+1.9}{x_{4}}-1\leq 0,\\ g_{11}\left(\vec{x}\right)=\frac{1.1x_{7}+1.9}{x_{5}}-1\leq 0, \end{array} \end{array}\right\}$$

Parameters range:

(25) $$\left.\begin{array}{c} \begin{array}{c} 2.6\leq x_{1}\leq 3.6,\\ 0.7\leq x_{2}\leq 0.8,\\ 17\leq x_{3}\leq 28,\\ 7.3\leq x_{4}\leq 8.3,\\ 7.8\leq x_{5}\leq 8.3,\\ 2.9\leq x_{6}\leq 3.9,\\ 5.0\leq x_{7}\leq 5.5, \end{array} \end{array}\right\}$$

The experimental results of the reducer design problem are presented in Table 9. From the average value, MDBO exhibits slightly superior performance compared to SSA and significantly outperforms other algorithms, underscoring its efficacy in achieving high solution accuracy for this problem. Additionally, considering the standard deviation, MDBO showcases the lowest value, indicating its exceptional stability and robustness in producing consistent results across multiple runs.

### 5.3. Welded Beam Design Issues

**Figure 13:**
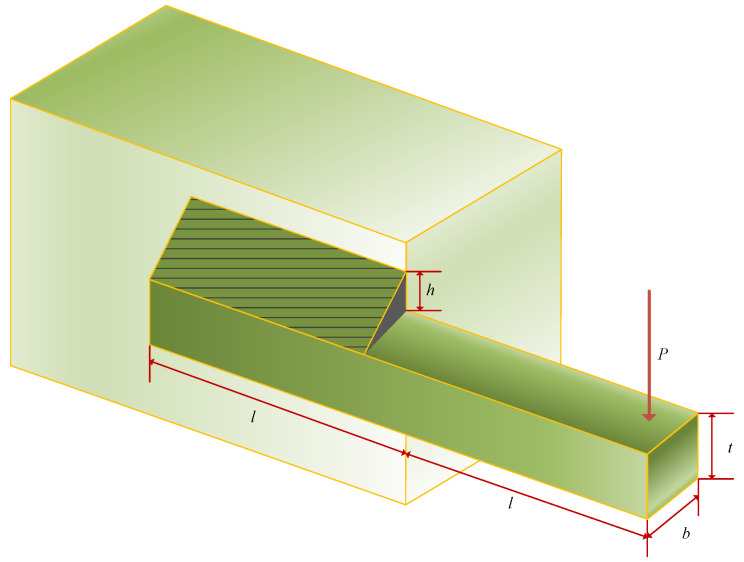
Welded beam design issues.

The objective of the welded beam design problem is to minimize the cost of the welded beam. As shown in Figure 13, the welded beam design problem exists with four parametric variables: Weld thickness (*h*), length of the connected portion of the bar (*l*), height of the bar (*t*), and thickness of the reinforcement bar (*b*) as in Equation (26). The objective function is defined in Equation (27), and its minimization process is bounded by the constraints of shear stresses (τ), bending stresses in the beam (θ), flexural loads on the bar ($P_{c}$), and end disturbances in the beam (δ) as in Equation (28). The four variable parameters are bounded as in Equation (29), and the values of certain parameters and their solutions are as Equation (30).

Consider:

(26) $$\tilde{x}=\left[x_{1},x_{2},x_{3},x_{4}\right]=\left[h,l,t,b\right],$$

Minimize:

(27) $$f\left(\vec{x}\right)=1.10471x_{1}^{2}x_{2}+0.04811x_{3}x_{4}\left(14.0+x_{2}\right),$$

Subject to:

(28) $$\left.\begin{array}{c} \begin{array}{c} g_{1}\left(\vec{x}\right)=\tau \left(\vec{x}\right)-\tau_{\max}\leq 0\\ g_{2}\left(\vec{x}\right)=\sigma \left(\vec{x}\right)-\tau_{\max}\leq 0\\ g_{3}\left(\vec{x}\right)=\delta \left(\vec{x}\right)-\tau_{\max}\leq 0\\ g_{4}\left(\vec{x}\right)=x_{1}-x_{4}\leq 0\\ g_{5}\left(\vec{x}\right)=P-P_{c}\left(\vec{x}\right)\leq 0\\ g_{6}\left(\vec{x}\right)=0.125-x_{1}\leq 0\\ g_{7}\left(\vec{x}\right)=1.10471x_{1}^{2}+0.04811x_{3}x_{4}\left(14.0+x_{2}\right)-5.0\leq 0 \end{array} \end{array}\right\}$$

Parameters range:

(29) $$0.1\leq x_{1},x_{4}\leq 2,0.1\leq x_{2},x_{3}\leq 10,$$

where

(30) $$\left.\begin{array}{c} \begin{array}{c} \tau \left(\vec{x}\right)=\sqrt{{\left(\tau^{\prime }\right)}^{2}+2\tau^{\prime }\tau^{\prime \prime }\frac{x_{2}}{2R}+{\left(\tau^{\prime }\right)}^{2}},\\ \tau^{\prime }=\frac{P}{\sqrt{2x_{1}x_{2}}},\tau^{\prime \prime }=\frac{MR}{J},\\ M=P(L+\frac{x_{2}}{2}),\\ R=\sqrt{\frac{x_{2}^{2}}{4}+{\left(\frac{x_{1}+x_{3}}{2}\right)}^{2}},\\ J=2(\sqrt{2}x_{1}x_{2}\left(\frac{x_{2}^{2}}{12}+{\left(\frac{x_{1}+x_{3}}{2}\right)}^{2}\right)),\\ \sigma \left(\vec{x}\right)=\frac{6PL}{x_{4}x_{3}^{2}},\delta \left(x\right)=\frac{4PL^{3}}{Ex_{3}^{3}x_{4}},\\ P_{c}\left(x\right)=\frac{4.013E\sqrt{\frac{x_{3}^{2}x_{4}^{6}}{36}}}{L^{2}}\left(1-\frac{x_{3}}{2L}\sqrt{\frac{E}{4G}}\right),\\ P=6000lb,L=14in,\delta_{\max}=0.25in,\\ E=30\times 10^{6}psi,G=12\times 10^{6}psi,\\ \tau_{\max}=13600psi,\sigma_{\max}=30000psi. \end{array} \end{array}\right\}$$

The optimization results for the welded beam design problem are displayed in Table 10. It is evident that MDBO achieves the lowest average manufacturing cost, with a value of 1.692769435 when the optimization result of MDBO is *x* = [0.205729953, 3.234915914, 9.036617034, 0.205729953]. When compared with other algorithms, MDBO demonstrates competitive performance, highlighting its effectiveness in this particular optimization task.

## 6. Conclusions

In this paper, based on the deficiencies of the DBO algorithm, the multi-strategy improved DBO algorithm (MDBO) is proposed. Firstly, Latin hypercube sampling is used to initialize the population to improve the diversity of the population and reduce the possibility of the algorithm falling into local optimal solutions. Second, mean difference variation is introduced to the population individuals to balance the local and global exploration of the algorithm and improve the algorithm’s ability to escape from the local optimum. Finally, fusion lens imaging back learning and dimension-by-dimension optimization are performed on the global optimal solution to make full use of the optimal solution information while improving the quality of the optimal solution and promoting the convergence of the algorithm. To verify the performance of the MDBO, this paper evaluates the performance of the algorithm from several aspects using the CEC2017 and CEC2020 test functions. Finally, the proposed MDBO algorithm is successfully applied to three real-world engineering application problems.

Through a large number of experimental results in several aspects, the proposed MDBO algorithm exhibits stronger optimization ability, faster convergence speed, higher convergence accuracy, and better robustness than other classical meta-heuristic algorithms, and it also demonstrates better performance in some engineering practical applications. However, MDBO still faces challenges in obtaining the theoretical optimum when solving some complex problems in a short time. In future work, on the one hand, some other novel algorithms can be combined to improve the efficiency and optimization ability of the algorithm; on the other hand, the optimized algorithm can be used to solve more complex optimization problems in reality, such as the UAV path planning, polling system [45,46], and the NP-hard problem.

## Figures and Tables

**Table 1:** CEC2017 and CEC 2020 functions.

Type	Function	Dimension	Minimum	CEC Type
Unimodal functions	Shifted and Rotated Bent Cigar Function	30D, 100D	100	CEC 2017 F1
	Shifted and Rotated Zakharov Function	30D, 100D	200	CEC 2017 F2
	Shifted and Rotated Bent Cigar Function	20D	100	CEC 2020 F1
Simple multimodal	Shifted and Rotated Rosenbrock’s Function	30D, 100D	300	CEC 2017 F3
	Shifted and Rotated Rastrigin’s Function	30D, 100D	400	CEC 2017 F4
	Shifted and Rotated Expanded Scaffer’s F6 Function	30D, 100D	500	CEC 2017 F5
	Shifted and Rotated Lunacek Bi_Rastrigin Function	30D, 100D	600	CEC 2017 F6
	Shifted and Rotated Non-Continuous Rastrigin’s Function	30D, 100D	700	CEC 2017 F7
	Shifted and Rotated Lecy Function	30D, 100D	800	CEC 2017 F8
	Shifted and Rotated Schwefel’s Function	30D, 100D	900	CEC 2017 F9
Basic functions	Shifted and Rotated Schwefel’s Function	20D	700	CEC 2020 F2
	Shifted and Rotated Lunacek Bi_Rastrigin Function	20D	1900	CEC 2020 F3
	Expanded Rosenbrock’s plus Griewangk’s Function	20D	1700	CEC 2020 F4
Hybrid functions	Hybrid Function 1 (N = 3)	30D, 100D	1000	CEC 2017 F10
	Hybrid Function 2 (N = 3)	30D, 100D	1100	CEC 2017 F11
	Hybrid Function 3 (N = 3)	30D, 100D	1200	CEC 2017 F12
	Hybrid Function 4 (N = 4)	30D, 100D	1300	CEC 2017 F13
	Hybrid Function 5 (N = 4)	30D, 100D	1400	CEC 2017 F14
	Hybrid Function 6 (N = 4)	30D, 100D	1500	CEC 2017 F15
	Hybrid Function 6 (N = 5)	30D, 100D	1600	CEC 2017 F16
	Hybrid Function 6 (N = 5)	30D, 100D	1700	CEC 2017 F17
	Hybrid Function 6 (N = 5)	30D, 100D	1800	CEC 2017 F18
	Hybrid Function 6 (N = 6)	30D, 100D	1900	CEC 2017 F19
	Hybrid Function 1 (N = 3)	20D	1700	CEC 2020 F5
	Hybrid Function 2 (N = 4)	20D	1600	CEC 2020 F6
	Hybrid Function 3 (N = 5)	20D	2100	CEC 2020 F7
Composition functions	Composition Function 1 (N = 3)	30D, 100D	2000	CEC 2017 F20
	Composition Function 2 (N = 3)	30D, 100D	2100	CEC 2017 F21
	Composition Function 3 (N = 4)	30D, 100D	2200	CEC 2017 F22
	Composition Function 4 (N = 4)	30D, 100D	2300	CEC 2017 F23
	Composition Function 5 (N = 5)	30D, 100D	2400	CEC 2017 F24
	Composition Function 6 (N = 5)	30D, 100D	2500	CEC 2017 F25
	Composition Function 7 (N = 6)	30D, 100D	2600	CEC 2017 F26
	Composition Function 7 (N = 6)	30D, 100D	2700	CEC 2017 F27
	Composition Function 9 (N = 3)	30D, 100D	2800	CEC 2017 F28
	Composition Function 10 (N = 3)	30D, 100D	2900	CEC 2017 F29
	Composition Function 1 (N = 3)	20D	2200	CEC 2020 F8
	Composition Function 2 (N = 4)	20D	2400	CEC 2020 F9
	Composition Function 3 (N = 5)	20D	2500	CEC 2020 F10
Search range: [−100,100]^*D*^				

**Table 2:** CEC2017 dimension for 30 test results.

		MDBO	DBO	WOA	GWO	SCA	SSA	HHO
	min	2.83E+02	8.37E+07	2.64E+09	4.48E+08	1.22E+10	**1.70E+02**	1.43E+08
F1	mean	1.57E+04	3.42E+08	5.36E+09	2.46E+09	2.04E+10	**6.43E+03**	4.77E+08
	std	3.61E+04	2.67E+08	2.11E+09	1.53E+09	3.95E+09	**6.19E+03**	2.79E+08
	min	**1.89E+04**	6.92E+04	1.21E+05	4.69E+04	5.35E+04	3.33E+04	4.11E+04
F2	mean	**3.22E+04**	9.73E+04	2.84E+05	6.55E+04	8.74E+04	5.06E+04	5.64E+04
	std	**7.14E+03**	4.07E+04	7.69E+04	1.22E+04	1.73E+04	7.85E+03	7.67E+03
	min	**4.70E+02**	5.31E+02	6.74E+02	5.10E+02	1.61E+03	4.30E+02	5.52E+02
F3	mean	**5.01E+02**	6.71E+02	1.53E+03	6.40E+02	2.93E+03	5.06E+02	7.58E+02
	std	**1.58E+01**	1.52E+02	6.60E+02	1.39E+02	7.80E+02	2.49E+01	1.43E+02
	min	**5.57E+02**	6.48E+02	7.35E+02	5.77E+02	7.80E+02	6.46E+02	6.93E+02
F4	mean	**6.00E+02**	7.52E+02	8.56E+02	6.24E+02	8.20E+02	7.46E+02	7.76E+02
	std	**2.73E+01**	5.55E+01	4.77E+01	3.58E+01	2.24E+01	5.58E+01	3.35E+01
	min	6.10E+02	6.31E+02	6.59E+02	**6.04E+02**	6.47E+02	6.22E+02	6.54E+02
F5	mean	6.17E+02	6.50E+02	6.76E+02	**6.12E+02**	6.65E+02	6.48E+02	6.67E+02
	std	4.68E+00	9.32E+00	1.01E+01	**4.11E+00**	8.43E+00	1.08E+01	6.26E+00
	min	**8.01E+02**	8.86E+02	1.12E+03	8.14E+02	1.18E+03	1.04E+03	1.14E+03
F6	mean	**8.85E+02**	1.00E+03	1.30E+03	9.01E+02	1.26E+03	1.23E+03	1.32E+03
	std	**4.30E+01**	6.51E+01	9.88E+01	5.93E+01	6.29E+01	8.59E+01	6.01E+01
	min	**8.48E+02**	9.25E+02	9.66E+02	8.60E+02	1.05E+03	9.06E+02	9.48E+02
F7	mean	**8.90E+02**	1.03E+03	1.09E+03	9.00E+02	1.10E+03	9.77E+02	9.93E+02
	std	**2.66E+01**	4.78E+01	6.51E+01	2.47E+01	2.62E+01	2.59E+01	2.38E+01
	min	**1.14E+03**	3.10E+03	5.54E+03	1.24E+03	6.24E+03	3.92E+03	6.34E+03
F8	mean	**1.79E+03**	6.85E+03	1.20E+04	2.86E+03	8.65E+03	5.31E+03	8.56E+03
	std	**3.76E+02**	2.49E+03	3.31E+03	1.10E+03	1.75E+03	3.93E+02	1.16E+03
	min	**3.54E+03**	4.64E+03	6.40E+03	3.51E+03	7.83E+03	4.29E+03	4.79E+03
F9	mean	**5.07E+03**	6.53E+03	7.34E+03	5.14E+03	8.97E+03	5.30E+03	5.99E+03
	std	**6.92E+02**	1.13E+03	6.15E+02	1.30E+03	3.27E+02	5.14E+02	7.47E+02
	min	**1.16E+03**	1.35E+03	4.94E+03	1.36E+03	2.84E+03	1.20E+03	1.28E+03
F10	mean	**1.21E+03**	1.97E+03	1.06E+04	2.57E+03	4.46E+03	1.33E+03	1.60E+03
	std	**3.34E+01**	7.72E+02	4.43E+03	1.13E+03	1.22E+03	7.98E+01	1.59E+02
	min	1.85E+05	5.49E+06	6.25E+07	1.13E+07	1.31E+09	**1.73E+05**	9.26E+06
F11	mean	2.47E+06	6.00E+07	5.05E+08	1.14E+08	2.59E+09	**1.40E+06**	8.78E+07
	std	2.89E+06	7.06E+07	3.63E+08	9.79E+07	7.54E+08	**1.15E+06**	8.97E+07
	min	1.65E+03	2.28E+04	1.42E+06	4.75E+04	4.67E+08	**5.48E+03**	4.53E+05
F12	mean	6.09E+05	9.81E+06	1.48E+07	2.13E+07	1.22E+09	**1.76E+05**	2.07E+06
	std	1.53E+06	1.97E+07	1.71E+07	6.03E+07	7.62E+08	**8.08E+05**	4.18E+06
	min	**2.92E+03**	8.75E+03	5.49E+04	2.36E+04	7.79E+04	3.65E+03	1.66E+04
F13	mean	**4.47E+04**	4.14E+05	2.56E+06	7.38E+05	7.67E+05	5.23E+04	1.29E+06
	std	**4.56E+04**	6.55E+05	2.51E+06	8.95E+05	5.65E+05	4.35E+04	1.22E+06
	min	**1.71E+03**	3.38E+03	2.19E+05	2.22E+04	5.46E+06	2.12E+03	2.53E+04
F14	mean	**1.36E+04**	1.17E+05	5.22E+06	3.88E+06	5.59E+07	1.46E+04	1.54E+05
	std	**1.00E+04**	1.96E+05	8.26E+06	1.37E+07	4.20E+07	1.39E+04	6.03E+04
	min	**2.09E+03**	2.37E+03	2.99E+03	2.32E+03	3.57E+03	2.16E+03	3.01E+03
F15	mean	**2.68E+03**	3.25E+03	4.46E+03	2.71E+03	4.16E+03	2.89E+03	3.71E+03
	std	**3.14E+02**	4.60E+02	6.61E+02	3.62E+02	2.72E+02	4.21E+02	4.90E+02
	min	**1.80E+03**	2.19E+03	2.09E+03	1.80E+03	2.43E+03	1.99E+03	2.12E+03
F16	mean	**2.10E+03**	2.72E+03	2.74E+03	2.11E+03	2.84E+03	2.55E+03	2.66E+03
	std	**1.90E+02**	2.58E+02	3.18E+02	1.81E+02	2.13E+02	2.79E+02	3.61E+02
	min	**1.06E+05**	1.19E+05	2.64E+05	7.38E+04	3.43E+06	1.02E+05	1.56E+05
F17	mean	**4.92E+05**	3.91E+06	1.49E+07	2.49E+06	1.74E+07	6.74E+05	2.96E+06
	std	**3.49E+05**	5.61E+06	1.30E+07	2.79E+06	1.24E+07	7.25E+05	2.78E+06
	min	1.98E+03	2.57E+03	3.63E+05	1.80E+04	2.12E+07	**2.05E+03**	8.69E+04
F18	mean	1.41E+04	7.24E+06	2.60E+07	1.45E+06	1.10E+08	**1.13E+04**	1.79E+06
	std	1.50E+04	1.34E+07	2.53E+07	2.65E+06	9.34E+07	**1.36E+04**	1.68E+06
	min	**2.14E+03**	2.37E+03	2.40E+03	2.19E+03	2.61E+03	2.37E+03	2.41E+03
F19	mean	**2.43E+03**	2.76E+03	2.91E+03	2.51E+03	2.94E+03	2.74E+03	2.83E+03
	std	**2.20E+02**	2.14E+02	2.38E+02	1.79E+02	1.51E+02	2.39E+02	2.13E+02
	min	**2.34E+03**	2.46E+03	2.55E+03	2.36E+03	2.54E+03	2.44E+03	2.51E+03
F20	mean	**2.38E+03**	2.57E+03	2.64E+03	2.41E+03	2.61E+03	2.50E+03	2.59E+03
	std	**2.14E+01**	6.16E+01	5.41E+01	4.02E+01	3.01E+01	4.79E+01	5.17E+01
	min	**2.30E+03**	2.35E+03	3.20E+03	2.52E+03	4.55E+03	2.30E+03	5.14E+03
F21	mean	**2.30E+03**	4.62E+03	7.68E+03	4.94E+03	9.71E+03	5.46E+03	7.59E+03
	std	**6.67E+00**	2.50E+03	2.12E+03	1.98E+03	1.77E+03	2.49E+03	8.72E+02
	min	**2.70E+03**	2.87E+03	2.98E+03	2.73E+03	3.01E+03	2.77E+03	3.07E+03
F22	mean	**2.76E+03**	3.03E+03	3.15E+03	2.79E+03	3.08E+03	2.93E+03	3.29E+03
	std	**3.29E+01**	9.46E+01	9.99E+01	5.10E+01	4.57E+01	7.21E+01	1.30E+02
	min	**2.87E+03**	3.00E+03	3.07E+03	2.86E+03	3.17E+03	2.94E+03	3.25E+03
F23	mean	**2.92E+03**	3.19E+03	3.30E+03	2.99E+03	3.25E+03	3.08E+03	3.54E+03
	std	**3.76E+01**	1.00E+02	1.08E+02	7.86E+01	3.51E+01	8.70E+01	1.38E+02
	min	2.88E+03	2.91E+03	3.12E+03	2.93E+03	3.28E+03	**2.88E+03**	2.95E+03
F24	mean	2.91E+03	2.99E+03	3.26E+03	3.02E+03	3.58E+03	**2.89E+03**	3.01E+03
	std	2.10E+01	6.62E+01	9.69E+01	8.49E+01	2.44E+02	**1.46E+01**	4.24E+01
	min	**2.90E+03**	5.44E+03	5.40E+03	4.41E+03	7.08E+03	2.80E+03	6.65E+03
F25	mean	**4.71E+03**	7.05E+03	8.38E+03	5.07E+03	7.92E+03	5.59E+03	8.44E+03
	std	**6.29E+02**	8.53E+02	1.22E+03	5.34E+02	4.42E+02	1.41E+03	1.15E+03
	min	**3.21E+03**	3.25E+03	3.28E+03	3.23E+03	3.38E+03	3.22E+03	3.32E+03
F26	mean	**3.26E+03**	3.33E+03	3.45E+03	3.27E+03	3.58E+03	3.26E+03	3.65E+03
	std	**2.69E+01**	6.40E+01	1.02E+02	2.69E+01	8.63E+01	3.42E+01	2.13E+02
	min	3.21E+03	3.30E+03	3.45E+03	3.30E+03	4.17E+03	**3.20E+03**	3.34E+03
F27	mean	3.24E+03	3.62E+03	3.89E+03	3.51E+03	4.55E+03	**3.23E+03**	3.47E+03
	std	1.99E+01	6.90E+02	2.32E+02	1.42E+02	3.38E+02	**2.00E+01**	9.65E+01
	min	3.54E+03	3.75E+03	4.35E+03	**3.51E+03**	4.71E+03	3.79E+03	4.24E+03
F28	mean	3.87E+03	4.46E+03	5.49E+03	**3.83E+03**	5.24E+03	4.23E+03	5.09E+03
	std	2.01E+02	4.13E+02	7.93E+02	**1.86E+02**	3.20E+02	2.82E+02	4.76E+02
	min	**7.10E+03**	2.52E+04	9.58E+06	8.65E+05	9.55E+07	6.78E+03	7.14E+05
F29	mean	**2.53E+04**	2.45E+06	5.94E+07	1.27E+07	1.99E+08	3.53E+04	1.54E+07
	std	**3.50E+04**	3.97E+06	4.60E+07	9.80E+06	8.05E+07	8.69E+04	1.65E+07
	Total	21	0	0	2	0	6	0

**Table 3:** CEC2017 dimensions for 100 test results.

		MDBO	DBO	WOA	GWO	SCA	SSA	HHO
	min	4.26E+09	2.03E+10	8.72E+10	2.78E+10	1.84E+11	**2.33E+08**	3.78E+10
F1	mean	1.49E+10	8.51E+10	1.12E+11	5.39E+10	2.12E+11	**4.03E+08**	5.09E+10
	std	7.78E+09	7.00E+10	1.18E+10	9.82E+09	1.36E+10	**1.21E+08**	6.18E+09
	min	**3.01E+05**	3.40E+05	4.29E+05	4.11E+05	4.70E+05	3.25E+05	3.23E+05
F2	mean	**3.55E+05**	6.33E+05	8.93E+05	5.24E+05	5.96E+05	7.68E+05	3.60E+05
	std	**3.56E+04**	2.54E+05	1.50E+05	7.56E+04	7.93E+04	1.94E+05	8.43E+04
	min	1.12E+03	3.56E+03	1.38E+04	2.83E+03	3.66E+04	**9.27E+02**	6.35E+03
F3	mean	1.87E+03	1.97E+04	2.09E+04	5.98E+03	5.36E+04	**1.03E+03**	9.23E+03
	std	4.41E+02	2.01E+04	4.82E+03	1.82E+03	7.81E+03	**5.63E+01**	1.62E+03
	min	1.12E+03	1.32E+03	1.72E+03	**1.08E+03**	1.93E+03	1.30E+03	1.56E+03
F4	mean	1.31E+03	1.70E+03	1.98E+03	**1.26E+03**	2.07E+03	1.37E+03	1.68E+03
	std	8.34E+01	2.27E+02	1.17E+02	**1.40E+02**	5.63E+01	4.06E+01	5.20E+01
	min	6.38E+02	6.61E+02	6.88E+02	**6.41E+02**	6.95E+02	6.61E+02	6.85E+02
F5	mean	6.52E+02	6.77E+02	7.07E+02	**6.46E+02**	7.05E+02	6.65E+02	6.92E+02
	std	6.09E+00	1.07E+01	9.51E+00	**3.61E+00**	5.19E+00	2.25E+00	3.94E+00
	min	2.03E+03	2.31E+03	3.58E+03	**1.98E+03**	3.73E+03	2.67E+03	3.48E+03
F6	mean	2.40E+03	2.91E+03	3.82E+03	**2.23E+03**	4.18E+03	3.21E+03	3.77E+03
	std	1.60E+02	3.43E+02	1.42E+02	**1.50E+02**	2.78E+02	1.42E+02	1.15E+02
	min	1.43E+03	1.76E+03	2.21E+03	**1.42E+03**	2.26E+03	1.71E+03	2.01E+03
F7	mean	1.58E+03	2.21E+03	2.41E+03	**1.56E+03**	2.43E+03	1.84E+03	2.14E+03
	std	7.67E+01	2.24E+02	1.11E+02	**6.95E+01**	6.49E+01	4.97E+01	6.22E+01
	min	1.84E+04	6.06E+04	5.53E+04	2.24E+04	7.34E+04	**2.45E+04**	6.23E+04
F8	mean	2.65E+04	7.70E+04	7.87E+04	4.52E+04	9.21E+04	**2.55E+04**	6.97E+04
	std	4.32E+03	6.40E+03	1.58E+04	1.24E+04	1.21E+04	**6.55E+02**	4.42E+03
	min	1.71E+04	1.82E+04	2.65E+04	1.63E+04	3.19E+04	**1.45E+04**	2.24E+04
F9	mean	2.00E+04	2.81E+04	2.93E+04	2.06E+04	3.32E+04	**1.72E+04**	2.47E+04
	std	1.33E+03	4.97E+03	1.30E+03	5.22E+03	4.93E+02	**1.46E+03**	1.68E+03
	min	**2.10E+04**	1.46E+05	1.81E+05	6.84E+04	1.15E+05	5.45E+04	9.18E+04
F10	mean	**4.63E+04**	2.35E+05	3.20E+05	9.27E+04	1.74E+05	8.95E+04	1.47E+05
	std	**1.17E+04**	4.46E+04	1.35E+05	1.41E+04	3.58E+04	1.95E+04	3.37E+04
	min	1.02E+08	2.69E+09	1.60E+10	5.03E+09	7.31E+10	**7.31E+07**	6.63E+09
F11	mean	4.76E+08	7.29E+09	3.12E+10	1.23E+10	9.98E+10	**1.66E+08**	1.12E+10
	std	5.16E+08	2.71E+09	8.34E+09	5.65E+09	1.09E+10	**4.93E+07**	2.63E+09
	min	**9.06E+03**	4.18E+05	1.09E+09	8.02E+07	9.89E+09	2.71E+04	4.97E+07
F12	mean	**2.59E+04**	3.84E+08	3.22E+09	1.79E+09	1.77E+10	1.83E+05	3.18E+08
	std	**2.11E+04**	3.07E+08	1.71E+09	1.45E+09	3.29E+09	6.86E+05	2.19E+08
	min	1.11E+06	2.97E+06	9.74E+06	1.82E+06	1.57E+07	**6.54E+05**	2.80E+06
F13	mean	3.20E+06	2.07E+07	2.44E+07	1.07E+07	6.54E+07	**2.30E+06**	9.48E+06
	std	1.71E+06	1.15E+07	1.09E+07	6.29E+06	3.18E+07	**1.06E+06**	3.17E+06
	min	**2.98E+03**	1.65E+05	1.89E+08	3.47E+07	3.05E+09	7.07E+03	5.57E+06
F14	mean	**6.39E+03**	9.12E+07	5.10E+08	2.62E+08	6.06E+09	2.03E+04	2.05E+07
	std	**4.23E+03**	1.57E+08	2.40E+08	4.00E+08	1.64E+09	1.19E+04	2.43E+07
	min	**4.16E+03**	6.91E+03	1.12E+04	4.97E+03	1.29E+04	5.39E+03	8.92E+03
F15	mean	**6.32E+03**	9.50E+03	1.72E+04	6.66E+03	1.50E+04	6.56E+03	1.08E+04
	std	**8.81E+02**	1.67E+03	3.13E+03	7.15E+02	1.13E+03	6.33E+02	1.24E+03
	min	**3.88E+03**	6.38E+03	8.49E+03	4.26E+03	1.63E+04	5.06E+03	6.77E+03
F16	mean	**5.13E+03**	9.53E+03	2.00E+04	5.49E+03	8.59E+04	5.99E+03	8.26E+03
	std	**6.58E+02**	1.90E+03	1.73E+04	6.94E+02	1.15E+05	5.89E+02	1.27E+03
	min	1.43E+06	6.33E+06	5.21E+06	2.90E+06	5.36E+07	**1.07E+06**	2.30E+06
F17	mean	4.26E+06	2.75E+07	2.04E+07	1.10E+07	1.29E+08	**3.11E+06**	9.50E+06
	std	1.77E+06	1.59E+07	1.10E+07	8.00E+06	5.14E+07	**1.45E+06**	4.69E+06
	min	**2.20E+03**	1.04E+07	1.78E+08	1.48E+07	2.61E+09	3.03E+03	9.49E+06
F18	mean	**7.28E+03**	8.54E+07	6.26E+08	2.17E+08	5.23E+09	1.76E+04	5.00E+07
	std	**6.35E+03**	6.48E+07	3.80E+08	2.33E+08	1.26E+09	2.29E+04	3.15E+07
	min	**3.97E+03**	5.91E+03	6.23E+03	3.87E+03	7.25E+03	4.04E+03	5.09E+03
F19	mean	**5.08E+03**	7.15E+03	7.16E+03	5.47E+03	8.04E+03	5.90E+03	6.16E+03
	std	**4.13E+02**	7.67E+02	5.49E+02	1.04E+03	3.44E+02	6.93E+02	4.77E+02
	min	**2.90E+03**	3.57E+03	4.02E+03	2.97E+03	3.97E+03	3.37E+03	3.97E+03
F20	mean	**3.00E+03**	4.05E+03	4.50E+03	3.11E+03	4.20E+03	3.65E+03	4.40E+03
	std	**7.73E+01**	1.97E+02	1.97E+02	1.19E+02	8.33E+01	1.86E+02	2.37E+02
	min	2.13E+04	2.12E+04	2.89E+04	1.94E+04	3.40E+04	**1.71E+04**	2.42E+04
F21	mean	2.42E+04	2.90E+04	3.17E+04	2.42E+04	3.53E+04	**2.01E+04**	2.76E+04
	std	1.18E+03	4.78E+03	1.35E+03	5.30E+03	6.19E+02	**1.60E+03**	1.79E+03
	min	**3.32E+03**	4.28E+03	4.75E+03	3.51E+03	4.92E+03	3.93E+03	5.45E+03
F22	mean	**3.49E+03**	4.91E+03	5.34E+03	3.72E+03	5.21E+03	4.21E+03	5.86E+03
	std	**8.56E+01**	2.45E+02	2.28E+02	9.79E+01	1.40E+02	2.00E+02	3.09E+02
	min	**3.74E+03**	5.39E+03	6.10E+03	4.20E+03	6.47E+03	4.55E+03	7.00E+03
F23	mean	**3.94E+03**	6.09E+03	6.73E+03	4.49E+03	7.36E+03	5.20E+03	8.53E+03
	std	**9.16E+01**	4.65E+02	3.57E+02	1.84E+02	3.96E+02	3.69E+02	6.93E+02
	min	4.10E+03	5.19E+03	9.05E+03	5.66E+03	1.76E+04	**3.45E+03**	5.89E+03
F24	mean	4.72E+03	8.45E+03	1.10E+04	7.15E+03	2.25E+04	**3.68E+03**	6.79E+03
	std	4.59E+02	4.95E+03	1.05E+03	8.97E+02	2.87E+03	**7.96E+01**	5.15E+02
	min	**1.17E+04**	2.04E+04	3.21E+04	1.43E+04	3.60E+04	5.01E+03	2.90E+04
F25	mean	**1.33E+04**	2.63E+04	3.80E+04	1.77E+04	4.17E+04	2.14E+04	3.12E+04
	std	**1.88E+03**	3.57E+03	3.13E+03	1.42E+03	2.79E+03	6.56E+03	1.34E+03
	min	**3.54E+03**	4.03E+03	4.74E+03	3.80E+03	7.68E+03	3.60E+03	5.33E+03
F26	mean	**3.77E+03**	4.56E+03	6.01E+03	4.34E+03	8.53E+03	3.84E+03	7.16E+03
	std	**1.63E+02**	2.99E+02	8.97E+02	2.67E+02	4.51E+02	1.86E+02	1.24E+03
	min	3.82E+03	7.73E+03	1.18E+04	6.84E+03	2.36E+04	**3.71E+03**	7.49E+03
F27	mean	4.70E+03	1.88E+04	1.47E+04	9.14E+03	2.71E+04	**3.83E+03**	9.48E+03
	std	5.94E+02	5.81E+03	1.38E+03	1.31E+03	2.29E+03	**6.75E+01**	8.93E+02
	min	**6.61E+03**	8.16E+03	1.56E+04	7.53E+03	2.23E+04	6.58E+03	1.07E+04
F28	mean	**7.73E+03**	1.21E+04	2.21E+04	9.37E+03	3.54E+04	7.78E+03	1.33E+04
	std	**5.39E+02**	5.76E+03	5.72E+03	1.02E+03	1.00E+04	5.67E+02	1.40E+03
	min	**5.89E+04**	3.60E+07	1.01E+09	7.84E+07	9.50E+09	2.36E+05	3.05E+08
F29	mean	**4.79E+05**	2.73E+08	2.72E+09	1.38E+09	1.34E+10	7.52E+05	7.27E+08
	std	**2.68E+05**	1.66E+08	1.24E+09	1.10E+09	2.88E+09	3.87E+05	3.59E+08
	Total	15	0	0	4	0	10	0

**Table 4:** CEC2020 dimension for 20 test results.

		MDBO	DBO	WOA	GWO	SCA	SSA	HHO
	min	**1.28E+02**	9.95E+03	5.11E+08	4.62E+05	5.64E+09	1.17E+02	8.36E+06
F1	mean	**2.84E+03**	3.00E+07	1.37E+09	1.10E+09	8.84E+09	3.46E+03	3.44E+07
	std	**3.21E+03**	2.51E+07	6.48E+08	1.12E+09	1.95E+09	3.92E+03	2.88E+07
	min	**1.58E+03**	2.48E+03	3.37E+03	2.03E+03	4.85E+03	1.84E+03	2.68E+03
F2	mean	**2.65E+03**	3.62E+03	4.29E+03	2.90E+03	5.43E+03	2.97E+03	3.55E+03
	std	**4.94E+02**	6.04E+02	4.96E+02	4.55E+02	2.64E+02	4.56E+02	4.31E+02
	min	7.58E+02	7.81E+02	9.03E+02	**7.53E+02**	8.92E+02	7.94E+02	8.76E+02
F3	mean	8.08E+02	8.40E+02	9.72E+02	**7.80E+02**	9.49E+02	8.93E+02	9.39E+02
	std	2.96E+01	4.22E+01	4.13E+01	**2.00E+01**	2.50E+01	4.92E+01	3.37E+01
	min	**1.90E+03**	1.91E+03	1.95E+03	1.90E+03	2.57E+03	1.90E+03	1.92E+03
F4	mean	**1.91E+03**	1.94E+03	2.91E+03	2.05E+03	4.91E+03	1.91E+03	1.93E+03
	std	**4.44E+00**	5.39E+01	1.64E+03	4.75E+02	1.92E+03	4.26E+00	1.05E+01
	min	**1.91E+04**	1.60E+05	5.76E+05	5.33E+04	7.43E+05	6.47E+04	3.02E+04
F5	mean	**1.58E+05**	1.06E+06	2.93E+06	1.13E+06	2.54E+06	2.06E+05	8.70E+05
	std	**1.18E+05**	1.16E+06	1.66E+06	1.03E+06	1.33E+06	1.00E+05	6.93E+05
	min	**1.61E+03**	1.67E+03	2.06E+03	1.68E+03	2.17E+03	1.60E+03	1.95E+03
F6	mean	**1.75E+03**	2.26E+03	2.63E+03	2.07E+03	2.51E+03	1.98E+03	2.29E+03
	std	**1.25E+02**	2.99E+02	3.13E+02	2.25E+02	1.90E+02	2.30E+02	1.95E+02
	min	**1.21E+04**	1.38E+04	1.79E+05	1.05E+04	1.98E+05	9.53E+03	5.51E+04
F7	mean	**8.84E+04**	4.44E+05	2.33E+06	1.29E+05	9.11E+05	1.88E+05	5.01E+05
	std	**8.12E+04**	6.09E+05	2.76E+06	9.69E+04	6.53E+05	2.04E+05	4.42E+05
	min	**2.30E+03**	2.31E+03	2.39E+03	2.31E+03	2.95E+03	2.30E+03	2.32E+03
F8	mean	**2.30E+03**	2.67E+03	4.60E+03	3.22E+03	5.45E+03	3.64E+03	3.50E+03
	std	**1.00E+00**	8.82E+02	1.82E+03	1.04E+03	1.87E+03	1.61E+03	1.54E+03
	min	**2.82E+03**	2.90E+03	2.89E+03	2.83E+03	2.99E+03	2.84E+03	3.01E+03
F9	mean	**2.86E+03**	2.99E+03	3.04E+03	2.87E+03	3.03E+03	2.94E+03	3.21E+03
	std	**2.20E+01**	4.89E+01	7.28E+01	4.47E+01	2.10E+01	7.26E+01	1.31E+02
	min	**2.91E+03**	2.91E+03	3.01E+03	2.92E+03	3.11E+03	2.90E+03	2.96E+03
F10	mean	**2.95E+03**	2.98E+03	3.13E+03	3.00E+03	3.29E+03	2.97E+03	3.02E+03
	std	**3.52E+01**	5.58E+01	7.10E+01	6.65E+01	1.54E+02	3.23E+01	2.50E+01
	Total	9	0	0	1	0	0	0

**Table 5:** CEC2017 dimension for the 30 Wilcoxon rank sum test.

	DBO	WOA	GWO	SCA	SSA	HHO
F1	3.02E-11	3.02E-11	3.02E-11	3.02E-11	**2.28E-01**	3.02E-11
F2	1.01E-08	3.02E-11	**1.19E-01**	2.03E-09	1.64E-05	**1.91E-01**
F3	8.99E-11	3.02E-11	2.87E-10	3.02E-11	**7.73E-01**	3.69E-11
F4	3.69E-11	3.02E-11	4.23E-03	3.02E-11	4.08E-11	3.02E-11
F5	7.39E-11	3.02E-11	4.06E-02	3.02E-11	6.70E-11	3.02E-11
F6	3.83E-06	3.02E-11	**2.28E-01**	3.02E-11	1.96E-10	3.02E-11
F7	4.50E-11	3.02E-11	**1.26E-01**	3.02E-11	1.33E-10	3.02E-11
F8	3.69E-11	3.02E-11	2.75E-03	3.02E-11	3.02E-11	3.02E-11
F9	9.51E-06	6.07E-11	**1.67E-01**	3.02E-11	**8.24E-02**	8.29E-06
F10	3.02E-11	3.02E-11	3.02E-11	3.02E-11	1.60E-07	3.02E-11
F11	4.98E-11	3.02E-11	3.34E-11	3.02E-11	**3.79E-01**	4.08E-11
F12	5.07E-10	4.62E-10	3.47E-10	3.02E-11	3.34E-03	5.57E-10
F13	4.64E-05	6.12E-10	6.05E-07	2.87E-10	**9.00E-01**	1.29E-09
F14	2.20E-07	3.02E-11	6.07E-11	3.02E-11	**8.30E-01**	5.49E-11
F15	1.31E-08	3.02E-11	**9.59E-01**	3.02E-11	1.63E-02	3.02E-11
F16	4.57E-09	3.82E-10	**8.53E-01**	4.08E-11	5.09E-06	6.53E-08
F17	**5.37E-02**	6.52E-09	**3.11E-01**	6.12E-10	2.42E-02	9.21E-05
F18	1.70E-08	3.02E-11	5.49E-11	3.02E-11	**6.10E-01**	3.02E-11
F19	3.99E-04	1.85E-08	**6.10E-01**	1.55E-09	9.51E-06	8.48E-09
F20	3.69E-11	3.02E-11	4.21E-02	3.02E-11	2.61E-10	3.69E-11
F21	3.02E-11	3.02E-11	3.02E-11	3.02E-11	2.32E-02	3.02E-11
F22	5.49E-11	3.02E-11	4.51E-02	3.02E-11	3.16E-10	3.02E-11
F23	9.92E-11	3.02E-11	2.42E-02	3.02E-11	5.97E-09	3.02E-11
F24	7.38E-10	3.02E-11	3.02E-11	3.02E-11	3.03E-02	3.02E-11
F25	3.82E-10	3.02E-11	**5.37E-02**	3.02E-11	1.25E-05	3.02E-11
F26	3.09E-06	1.21E-10	**6.10E-01**	3.02E-11	1.27E-02	3.34E-11
F27	3.02E-11	3.02E-11	3.02E-11	3.02E-11	4.08E-05	3.02E-11
F28	6.12E-10	3.02E-11	**1.12E-01**	3.02E-11	3.83E-06	4.98E-11
F29	1.09E-10	3.02E-11	3.02E-11	3.02E-11	**6.73E-01**	3.02E-11
Total	28	29	18	29	21	28

**Table 6:** CEC2017 dimension for 100 Wilcoxon rank sum test.

	DBO	WOA	GWO	SCA	SSA	HHO
F1	2.15E-10	3.02E-11	4.50E-11	3.02E-11	3.02E-11	3.02E-11
F2	1.56E-08	3.34E-11	8.99E-11	3.02E-11	3.47E-10	**7.51E-01**
F3	3.02E-11	3.02E-11	3.34E-11	3.02E-11	3.34E-11	3.02E-11
F4	3.82E-10	3.02E-11	1.44E-03	3.02E-11	1.32E-04	3.02E-11
F5	6.70E-11	3.02E-11	2.60E-05	3.02E-11	1.69E-09	3.02E-11
F6	6.52E-09	3.02E-11	8.15E-05	3.02E-11	3.69E-11	3.02E-11
F7	3.02E-11	3.02E-11	**2.71E-01**	3.02E-11	3.69E-11	3.02E-11
F8	3.02E-11	3.02E-11	4.69E-08	3.02E-11	2.84E-01	3.02E-11
F9	1.73E-07	3.02E-11	3.03E-02	3.02E-11	1.85E-08	3.69E-11
F10	3.02E-11	3.02E-11	3.34E-11	3.02E-11	8.99E-11	3.02E-11
F11	3.02E-11	3.02E-11	3.02E-11	3.02E-11	1.17E-05	3.02E-11
F12	3.02E-11	3.02E-11	3.02E-11	3.02E-11	6.01E-08	3.02E-11
F13	2.87E-10	3.02E-11	1.60E-07	3.02E-11	4.84E-02	8.89E-10
F14	3.02E-11	3.02E-11	3.02E-11	3.02E-11	1.31E-08	3.02E-11
F15	1.61E-10	3.02E-11	**1.41E-01**	3.02E-11	**4.38E-01**	3.02E-11
F16	3.34E-11	3.02E-11	**7.48E-02**	3.02E-11	5.46E-06	3.02E-11
F17	4.50E-11	8.99E-11	3.65E-08	3.02E-11	1.03E-02	7.60E-07
F18	3.02E-11	3.02E-11	3.02E-11	3.02E-11	3.18E-03	3.02E-11
F19	3.02E-11	3.02E-11	**2.34E-01**	3.02E-11	1.86E-06	9.76E-10
F20	3.02E-11	3.02E-11	2.28E-05	3.02E-11	3.02E-11	3.02E-11
F21	1.34E-05	3.02E-11	1.44E-03	3.02E-11	9.92E-11	1.07E-09
F22	3.02E-11	3.02E-11	1.41E-09	3.02E-11	3.02E-11	3.02E-11
F23	3.02E-11	3.02E-11	3.02E-11	3.02E-11	3.02E-11	3.02E-11
F24	1.46E-10	3.02E-11	3.69E-11	3.02E-11	3.02E-11	3.02E-11
F25	5.49E-11	3.02E-11	1.07E-09	3.02E-11	1.73E-06	3.02E-11
F26	6.07E-11	3.02E-11	5.07E-10	3.02E-11	**5.19E-02**	3.02E-11
F27	3.02E-11	3.02E-11	3.02E-11	3.02E-11	2.37E-10	3.02E-11
F28	5.49E-11	3.02E-11	7.12E-09	3.02E-11	**9.47E-01**	3.02E-11
F29	3.02E-11	3.02E-11	3.02E-11	3.02E-11	4.03E-03	3.02E-11
Total	29	29	25	29	26	28

**Table 7:** CEC2020 dimension for 20 Wilcoxon rank sum test.

	DBO	WOA	GWO	SCA	SSA	HHO
F1	3.02E-11	3.02E-11	3.02E-11	3.02E-11	**7.96E-01**	3.02E-11
F2	1.87E-07	1.33E-10	1.91E-02	3.02E-11	5.32E-03	4.31E-08
F3	3.38E-04	9.17E-08	6.92E-07	6.80E-08	6.92E-07	6.80E-08
F4	6.67E-06	6.80E-08	7.90E-08	6.80E-08	**8.60E-01**	2.56E-07
F5	1.56E-08	3.02E-11	2.15E-06	3.02E-11	3.39E-02	2.38E-07
F6	2.06E-06	7.90E-08	9.17E-08	6.80E-08	1.61E-04	1.66E-07
F7	2.25E-04	8.15E-11	**8.77E-02**	6.70E-11	**5.55E-02**	1.07E-07
F8	6.01E-07	6.80E-08	6.80E-08	6.80E-08	**3.65E-01**	6.80E-08
F9	4.50E-11	4.08E-11	**9.47E-01**	3.02E-11	1.11E-06	3.02E-11
F10	6.10E-03	3.02E-11	5.97E-05	3.02E-11	**7.98E-02**	1.17E-09
Total	10	10	8	10	5	10

**Table 8:** Extension/compression spring design issues.

Algorithm	d	D	N	Cost	Mean	Std
MDBO	0.05205627	0.365616261	10.78572664	0.012667678	0.012912098	0.000699969
DBO	0.05	0.317155606	14.07383987	0.012744771	0.013790323	0.001855465
WOA	0.059038565	0.560664184	4.948552659	0.01357903	0.013545488	0.000955076
GWO	0.050283697	0.323556018	13.57137641	0.012738869	0.012806882	0.00016799
SCA	0.05	0.314732431	14.56303889	0.013032314	0.013108942	0.000402296
SSA	0.05	0.317425416	14.02776975	0.012719054	0.013607165	0.001516753
HHO	0.061301593	0.635100129	3.957255992	0.014217786	0.013792769	0.001029114

**Table 9:** Reducer design issues.

Algorithm	x1	x2	X3	x4	x5	X6	x7	Cost	Mean	Std
MDBO	3.5000	0.7	17.0000	7.3000	7.8000	3.3502	5.2867	2996.3482	2996.3482	0.0000
DBO	3.5000	0.7	17.0000	8.3000	8.3000	3.3522	5.2869	3016.7704	3031.6754	59.1161
WOA	3.6000	0.7	18.4408	7.5166	8.2579	3.3495	5.4936	3450.3018	3427.3548	612.9289
GWO	3.5067	0.7	17.0000	8.2896	8.0026	3.3603	5.2898	3016.8089	3010.8418	4.3596
SCA	3.6000	0.7	17.0000	7.7987	8.3000	3.5246	5.2967	3104.4867	3127.8248	43.4361
SSA	3.5000	0.7	17.0000	7.3000	7.8000	3.3502	5.2867	2996.3482	2996.6593	1.7041
HHO	3.5121	0.7	20.6747	7.3000	8.0287	3.3480	5.2905	3705.9316	3536.5022	454.2663

**Table 10:** Welded beam design issues.

Algorithm	h	l	t	d	Cost	Mean	Std
MDBO	0.205729953	3.234915914	9.036617034	0.205729953	1.692769435	1.6961213	0.0127085
DBO	0.141565272	5.499670887	9.045400709	0.206123404	1.870870809	1.7496565	0.0424308
WOA	0.361675817	1.717131037	8.511619427	0.36198865	2.577921166	2.4658900	0.6156943
GWO	0.204259434	3.270735273	9.043281341	0.205752865	1.696780922	1.6983641	0.0030933
SCA	0.181314963	3.549003446	10	0.202491425	1.838490606	1.8835081	0.0628893
SSA	0.205729523	3.234915257	9.03663848	0.205729567	1.692769481	1.7833399	0.2636062
HHO	0.194218543	3.601102558	8.836192097	0.215168645	1.760035931	1.9306548	0.1725821

## Data Availability

All data are contained within the article.
